# A Comparative Study of Adaptive Interlimb Coordination Mechanisms for Self-Organized Robot Locomotion

**DOI:** 10.3389/frobt.2021.638684

**Published:** 2021-04-12

**Authors:** Tao Sun, Xiaofeng Xiong, Zhendong Dai, Dai Owaki, Poramate Manoonpong

**Affiliations:** ^1^Institute of Bioinspired Structure and Surface Engineering, College of Mechanical and Electrical Engineering, Nanjing University of Aeronautics and Astronautics, Nanjing, China; ^2^Embodied Artificial Intelligence and Neurorobotics Laboratory, SDU Biorobotics, The Mærsk Mc-Kinney Møller Institute, University of Southern Denmark, Odense, Denmark; ^3^Department of Robotics, Graduate School of Engineering, Tohoku University, Sendai, Japan

**Keywords:** adaptive interlimb coordination, phase resetting, phase modulation, decoupled CPGs, sensory feedback, self-organized locomotion

## Abstract

Walking animals demonstrate impressive self-organized locomotion and adaptation to body property changes by skillfully manipulating their complicated and redundant musculoskeletal systems. Adaptive interlimb coordination plays a crucial role in this achievement. It has been identified that interlimb coordination is generated through dynamical interactions between the neural system, musculoskeletal system, and environment. Based on this principle, two classical interlimb coordination mechanisms (continuous phase modulation and phase resetting) have been proposed independently. These mechanisms use decoupled central pattern generators (CPGs) with sensory feedback, such as ground reaction forces (GRFs), to generate robot locomotion autonomously without predefining it (i.e., self-organized locomotion). A comparative study was conducted on the two mechanisms under decoupled CPG-based control implemented on a quadruped robot in simulation. Their characteristics were compared by observing their CPG phase convergence processes at different control parameter values. Additionally, the mechanisms were investigated when the robot faced various unexpected situations, such as noisy feedback, leg motor damage, and carrying a load. The comparative study reveals that the phase modulation and resetting mechanisms demonstrate satisfactory performance when they are subjected to symmetric and asymmetric GRF distributions, respectively. This work also suggests a strategy for the appropriate selection of adaptive interlimb coordination mechanisms under different conditions and for the optimal setting of their control parameter values to enhance their control performance.

## 1. Introduction

Walking animals demonstrate impressive self-organized locomotion and adaptation to body property changes by skillfully manipulating their complicated and redundant musculoskeletal systems (Taga et al., 1991; Dickinson et al., 2000; Der and Martius, 2012; Grabowska et al., 2012). Many studies have clarified that adaptive interlimb coordination plays a crucial role in this achievement (Aoi et al., 2017; Mantziaris et al., 2017). Investigations of various aspects of adaptive interlimb coordination mechanisms have attracted significant attention in various research fields.

To demonstrates these mechanisms, biologists have proposed some neurological principles, such as central pattern generators (CPGs) (Marder and Bucher, 2001), reflex chains (Grillner, 1975), and sensory feedback (Grillner, 2003; Rossignol et al., 2006), through biological experiments. In addition, roboticists have developed many bio-inspired neural control schemes for legged robots to emulate animal-like self-organized locomotion (Kimura et al., 2007; Owaki et al., 2013; Barikhan et al., 2014; Ambe et al., 2018; Fukui et al., 2019; Miguel-Blanco and Manoonpong, 2020). To realize self-organized locomotion and adaptation on artificial legged systems, many adaptive robot control schemes based on distributed abstract CPGs incorporating ground reaction force (GRF) feedback have been proposed (Kimura et al., 2007; Owaki et al., 2013; Barikhan et al., 2014; Ambe et al., 2018; Fukui et al., 2019). Specifically, the GRF feedback is exploited to modulate the phase relationships of the CPGs under two main strategies: (continuous) phase modulation (PM) and (discrete) phase resetting (PR).

PM typically uses continuous GRFs to modulate CPG phases continuously (Kimura et al., 2007; Owaki et al., 2013, 2017; Barikhan et al., 2014; Fukuhara et al., 2018; Miguel-Blanco and Manoonpong, 2020). In contrast, the PR uses discrete GRFs to reset the CPG phases intermittently (Tsujita et al., 2001; Aoi and Tsuchiya, 2007; Nomura et al., 2009; Aoi et al., 2010, 2012; Ambe et al., 2018). While both mechanisms have proved their effectiveness in their own right and have been widely used in various fashions, they have not been systematically analyzed and compared to identify their characteristics in detail. For instance, how the control parameter values of the mechanisms influence the phase convergence process and whether the mechanisms show different performances in different situations. It is necessary to consider in which situations the PM (PR) works better.

**Figure 1:**
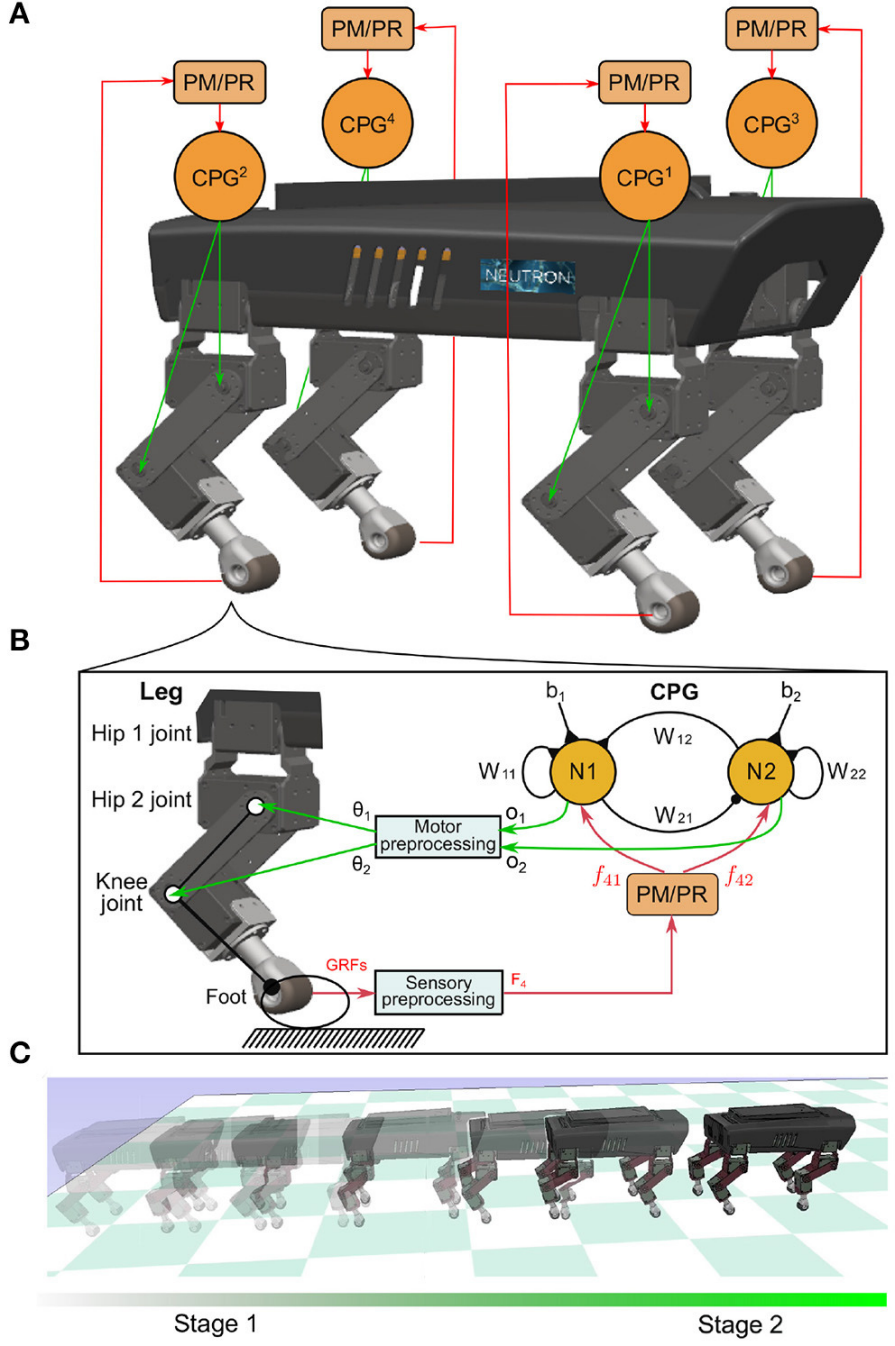
**(A)** Four identical and decoupled neural SO (2)-based CPGs modulated by the PM or PR relying on the sensory feedback (i.e., GRFs). They are used to control a quadruped robot. **(B)** Each CPG is composed of two mutually connected neurons. It outputs two synchronized signals (*o*_1,2_). The signals are linearly re-scaled as motor commands (θ_1,2_) for controlling the hip 2 and knee joints of a leg through the motor preprocessing unit. For simplicity, here the hip 1 joint is kept fixed and set to a certain position. **(C)** The quadruped was demonstrated under the self-organized locomotion generation process. The process was divided into two stages: transition (Stage 1) and formation (Stage 2).

From this point of view, a comparative study of the PM and PR for self-organized locomotion was conducted. They were used to modulate four decoupled neural SO (2)-based CPGs^1^ (Pasemann et al., 2003) relying on local GRF information. The modulated CPGs, acting as an adaptive neural controller, were implemented on a quadruped robot in simulation, as shown in Figures 1A,B. The CPG outputs were utilized to drive the robot joint movements such that the robot could autonomously perform self-organized locomotion, as shown in Figure 1C. The study focused on: (1) the parameter characteristics of the PM and PR and (2) their adaptations to unexpected robot situations (e.g., noisy feedback, leg motor damage, and carrying a load). The validation of the study was quantified by three metrics including: phase convergence time, phase deviation, and cost of transport (COT). Consequently, this work provides suggestions on how to choose adaptive interlimb coordination mechanisms properly in different situations and set their control parameter values optimally to enhance their control performance.

The rest of this article is structured as follows. Details of the materials and methods are provided in section 2. The experimental results are presented in section 3. A discussion of the experimental results and the conclusions are provided in section 4.

## 2. Materials and Methods

In this section, the adaptive neural controller for studying the PM and PR is elucidated. It is composed of four identical and decoupled neural SO (2)-based CPGs (Pasemann et al., 2003; Sun et al., 2018) modulated by the PM or PR. Subsequently, a simulation environment with a quadruped robot (called “Lilibot”) is introduced. It is an experimental platform for assessing the PM and PR by implementing the adaptive neural controller on the robot to generate self-organized locomotion. In addition, certain variables and metrics for analyzing and assessing the CPG phase convergence and self-organized locomotion are introduced.

### 2.1. Adaptive Neural Controller

The adaptive neural controller integrates the four CPGs with either PM or PR. The controller was proposed for easily demonstrating the PM and PR in an integrative manner. The PM and PR have numerous forms that comply with different CPG models and robots (Kimura et al., 2007; Owaki et al., 2013; Barikhan et al., 2014; Sun et al., 2020). To compare the PM and PR conveniently and consistently, four neural SO (2) oscillators are used as four decoupled CPGs. The SO (2)-based CPG has a simple neural network topology with analyzable neural dynamics (Pasemann et al., 2003). Thus, it can easily integrate either the PM or the PR for straightforwardly modulating or resetting the CPG' phase. Detailed descriptions are provided in the following.

#### 2.1.1. Decoupled Neural SO (2)-Based CPGs

Four decoupled neural SO (2)-based CPGs were used to produce multiple periodic signals for driving the quadruped robot (see Figure 1). Each neural SO (2)-based CPG consists of two connected neurons, where their neural activities are later adjusted by the PM or PR. It outputs two periodic signals that are transferred by a motor preprocessing unit to drive the hip 2 and knee joints of a leg. As a result, the leg's foot can trace a proper ellipse-like trajectory with swing forward and stance backward. The foot movement status detected by the GRF is transferred to the PM or PR through a sensory preprocessing unit. Based on the GRF feedback, the PM or PR generates modulation signals to its corresponding CPG. In the single closed-loop CPG-based control, the outputs of the CPG coordinate the two joint movements of the leg (i.e., intralimb coordination), while the interlimb coordination among legs is realized only by the interactions between the robot body dynamics and the environment (i.e., physical communications) through the PM (Owaki et al., 2013) or PR (Aoi et al., 2012) with GRF feedback of each leg. This is because the four CPGs are decoupled and have no direct neural communication between them. The four CPGs can be described using a matrix in discrete time equations as follows:

(1) $$\begin{array}{r} a\left(n+1\right)=w\cdot o\left(n\right)+b+f\left(n\right) \end{array}$$

(2) $$\begin{array}{r} o=\tanh\left(a\right), \end{array}$$

where ***a*** = (*a*_*ik*_), ***o*** = (*o*_*ik*_), and $b=\left(b_{ik}\right)\in {\mathbb{R}}^{2\times 4}$ represent the activations, outputs and biases of the CPG neurons, respectively. Each column of the three matrix variables (i.e., ***a***, ***o***, and ***b***) represents the values of a CPG. Moreover, *n* indicates the time of the discrete-time equations, where the update frequency is 60 Hz in the following investigations. ***w*** ∈ ℝ^2×2^ is the synaptic weights of a CPG (see Equation 4). $f=\left(f_{ik}\right)\in {\mathbb{R}}^{2\times 4}$ represents the modulation term of the PM or PR (see Equations 6–8). *f*_*ik*_ is the PM or PR term projecting to the *i*th neuron of the *k*th CPG. The projection can adjust the CPG neuron activities online, thereby resulting in the CPG phase adaptation.

The CPG outputs (***o***) are used to drive the joint movements through a linear transformation of the motor preprocessing unit (see Figure 1). It is given by the following equation:

(3) $$\begin{array}{r} \theta =\alpha o+\beta , \end{array}$$

where ***θ*** and ***β*** ∈ ℝ^2×4^ represent the desired joint angles and their biases, respectively.

Based on previous work (Manoonpong et al., 2013), each SO (2)-based CPG can generate periodic coordinated signals for intralimb and interlimb coordination by setting its weights and biases as follows:

(4) $$w=\left(\begin{array}{cc} 1.4 & \;2.6\\ -2.6 & \;1.4 \end{array}\right),$$

(5) $$b=\left(\begin{array}{cccc} 0.01 & \;0.01 & \;0.01 & \;0.01\\ 0.01 & \;0.01 & \;0.01 & \;0.01 \end{array}\right).$$

The CPGs' parameter setup is used for the following investigations.

#### 2.1.2. Phase Modulation (PM) Mechanism

The fundamental principle of the PM is to modulate the CPG phase continuously by relying on the continuous GRF signal. Based on the model of the neural SO (2)-based CPG with sensory feedback introduced by (Barikhan et al., 2014), a modified version of the sensory feedback is proposed. It is formulated as the PM modulation term in the following equations:

(6) $$f_{ik}(n)=\{\begin{array}{c} -\gamma \frac{F_{k}(n)}{mg}\cos(o_{ik}(n)),\;i=1,\\ -\gamma \frac{F_{k}(n)}{mg}\sin(o_{ik}(n)),\;i=2, \end{array}$$

where *o*_*ik*_ is the output of the *i*th neuron in the *k*th CPG, γ is a positive constant that represents the sensory feedback gain, and *F*_*k*_ is the GRF value whose range depends on the specific robot weight. Here, *mg* represents the weight of the robot. It is 2.5 kg for the robot used in the investigations. The robot weight is introduced to normalize the sensory feedback gain for generalization. In addition, γ is a dimensionless parameter that is independent of the robot.

From Equation (6), one can find that the greater the *F*_*k*_(*n*) a leg perceives, the higher the inhibition [if *f*_*ik*_(*n*) < 0] or excitation [if *f*_*ik*_(*n*) > 0] the corresponding leg's PM makes. More specifically, when the robot is on the ground, its four legs support and promote the robot body together. Thus, there is an approximately equal distribution among the GRFs of the four legs during locomotion. This means that, when the GRF of a stance leg decreases, the GRFs of other stance legs must increase. Therefore, the four CPGs have different modulation strengths. This results in phase differences among the four CPGs. Once the CPG phase differences converge to a proper status, adaptive interlimb coordination (i.e., self-organized locomotion) emerges (Owaki et al., 2013; Sun et al., 2018).

#### 2.1.3. Phase Resetting (PR) Mechanism

The fundamental principle of the PR is to reset the CPG phase intermittently by relying on the discrete GRF signal. For neural SO (2)-based CPG, the PR functionality is realized by resetting the CPG neuron activities to specific values when the GRF value increases over a threshold. Thus, the PR modulation term can be described as follows:

(7) $$f_{ik}(n)=\{\begin{array}{c} (1-(w_{11}o_{1k}(n)+w_{12}o_{2k}(n)+b_{1k}))\kappa ,\;i=1,\\ \;-(w_{21}o_{1k}(n)+w_{22}o_{2k}(n)+b_{2k})\kappa ,\;i=2, \end{array}$$

(8) $$\kappa =\{\begin{array}{l} 1.0,\;F_{k}(n)>F_{t}\frac{mg}{4},\;F_{k}(n-1)\;⩽\;F_{t}\frac{mg}{4}\\ 0.0,\text{ otherwise } \end{array},$$

where *o*_*ik*_ is the activity/output of the *i*th neuron in the *k*th CPG, *mg* is the weight of the robot, and *F*_*t*_ is a positive value representing GRF threshold factor that influences the timing of the PR. Here, $\frac{mg}{4}$ is regarded as a reference GRF value given that the four legs share the support of the robot weight. Once the GRF [*F*_*k*_(*n*)] of a leg becomes more than $\frac{mg}{4}$, the leg is indicated to be in the stance phase, thereby triggering the PR. Thus, to realize proper phase resetting, *F*_*t*_ value can be easily set in a small range ~1.0. Moreover, *F*_*t*_ is a dimensionless parameter that is independent of the robot.

More specifically, the condition in Equation (8) indicates that once the GRF value of a leg increases over $F_{t}\frac{mg}{4}$, then κ of the leg (e.g., the *k*th leg) is equal to 1.0. As a result,

(9) $$f_{ik}(n)=\{\begin{array}{l} 1-(w_{11}o_{1k}(n)+w_{12}o_{2k}(n)+0.01),\;i=1,\\ -(w_{21}o_{1k}(n)+w_{22}o_{2k}(n)+0.01),\;i=2, \end{array}.$$

Replacing them into Equations (1) and (2), the *k*th neural SO(2)-based CPG outputs at the next step are approximately reset to:

(10) $$\begin{array}{c} o_{ik}(n+1)=\tanh(a_{ik}(n+1))\\ =\{\begin{array}{l} \tanh(w_{11}o_{1k}(n)+w_{12}o_{2k}(n)+0.01+1-(w_{11}o_{1k}(n)+w_{12}o_{2k}(n)+0.01)),\;i=1,\\ \tanh(w_{21}o_{1k}(n)+w_{22}o_{2k}(n)+0.01-(w_{21}o_{1k}(n)+w_{22}o_{2k}(n)+0.01)),\;i=2, \end{array}\\ =\{\begin{array}{l} \tanh(1),\;i=1,\\ \tanh(0),\;i=2, \end{array}\\ \approx \{\begin{array}{l} 0.76,\;i=1,\\ 0,\;i=2, \end{array}. \end{array}$$

The CPG outputs are reset to the approximation from its limit cycle when a phase-resetting event occurs, followed by the CPG outputs returning to its limit cycle (see **Figure 3A**). Owing to the differences among the four GRFs, the phases of the CPGs are reset at different moments, thereby having phase differences. For example, when the robot wriggles with four legs supporting it on the ground, the GRFs of the four legs are close to $F_{t}\frac{mg}{4}$. In this case, the robot torso twisting back and forth leads to the GRFs with different change tendencies (e.g., front leg GRFs increase while hind leg GRFs decrease), which results in the GRFs of the legs meeting the PR condition at different moments. When the CPG phase differences converge to a proper status, adaptive interlimb coordination (i.e., self-organized locomotion) emerges (Aoi et al., 2010, 2012). More detailed information on the locomotion generation process can be found in the following experiments and corresponding videos.

### 2.2. Experimental Platform

**Figure 2:**
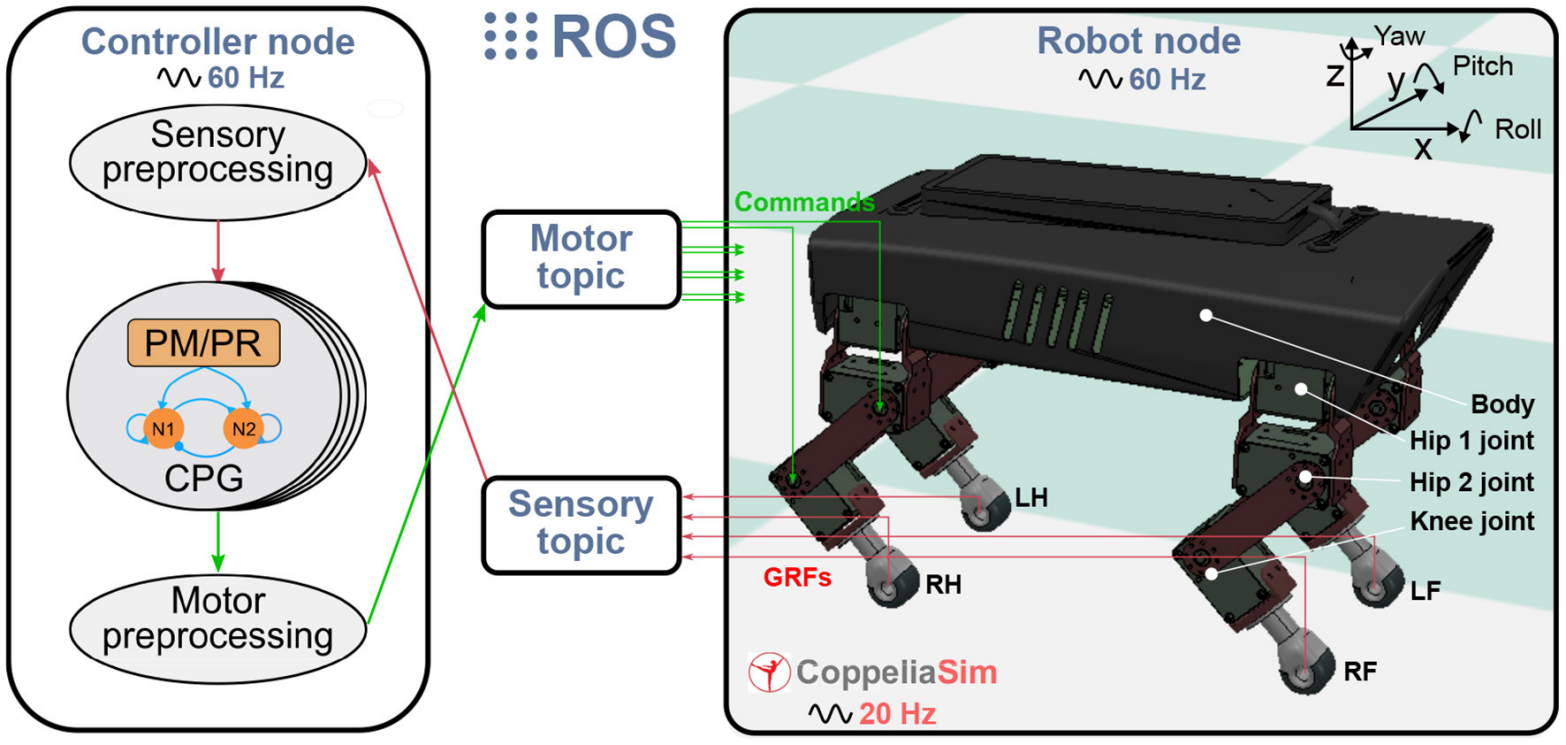
Experimental platform with the quadruped robot in CoppeliaSim (20 Hz) communication with the adaptive neural controller. The controller and the robot are regarded as two ROS nodes (60 Hz) and communicate with each other through two ROS topics. A motor topic transfers commands from the motor preprocessing unit of the controller node to the robot joints while a sensory topic acquires GRF signals from the robot and then send them to the sensory pre-processing unit of the controller node.

The experimental platform for studying the PM and PR is a quadruped robot in the simulation. The simulated robot is based on a small-size quadruped robot with multiple sensory feedback (Lilibot) which was developed in our previous works (Sun et al., 2020). The simulation environment was built using CoppeliaSim^2^ with physical engine Vortex^3^. The framework for connecting the robot with the adaptive neural controller (including the PM or PR) is based on the robot operation system (ROS)^4^ (see Figure 2). The robot and controller are regarded as two ROS nodes and communicate with each other through two ROS topics. A motor topic is used to transfer commands from the controller node to the robot node, while a sensory topic is used to acquire GRF signals from the robot node and then send them to the controller node. The update frequency of the two ROS nodes is 60 Hz, the CoppeliaSim calculation time step is 50 ms (20 Hz) during which main script of the simulated models is executed once. The simulation runs on a laptop (Thinkpad E470C) setup with an Intel Core i5-7200U and 8GB DDR4. The detailed information and source of the robotic platform can be found at https://gitlab.com/neutron-nuaa/lilibot. The launch sequence of the modules in the simulation is the CoppeliaSim initially and the two ROS nodes after 60 steps (3 s in CoppeliaSim).

### 2.3. Measurement of CPG Phase Convergence and Self-Organized Locomotion

**Figure 3:**
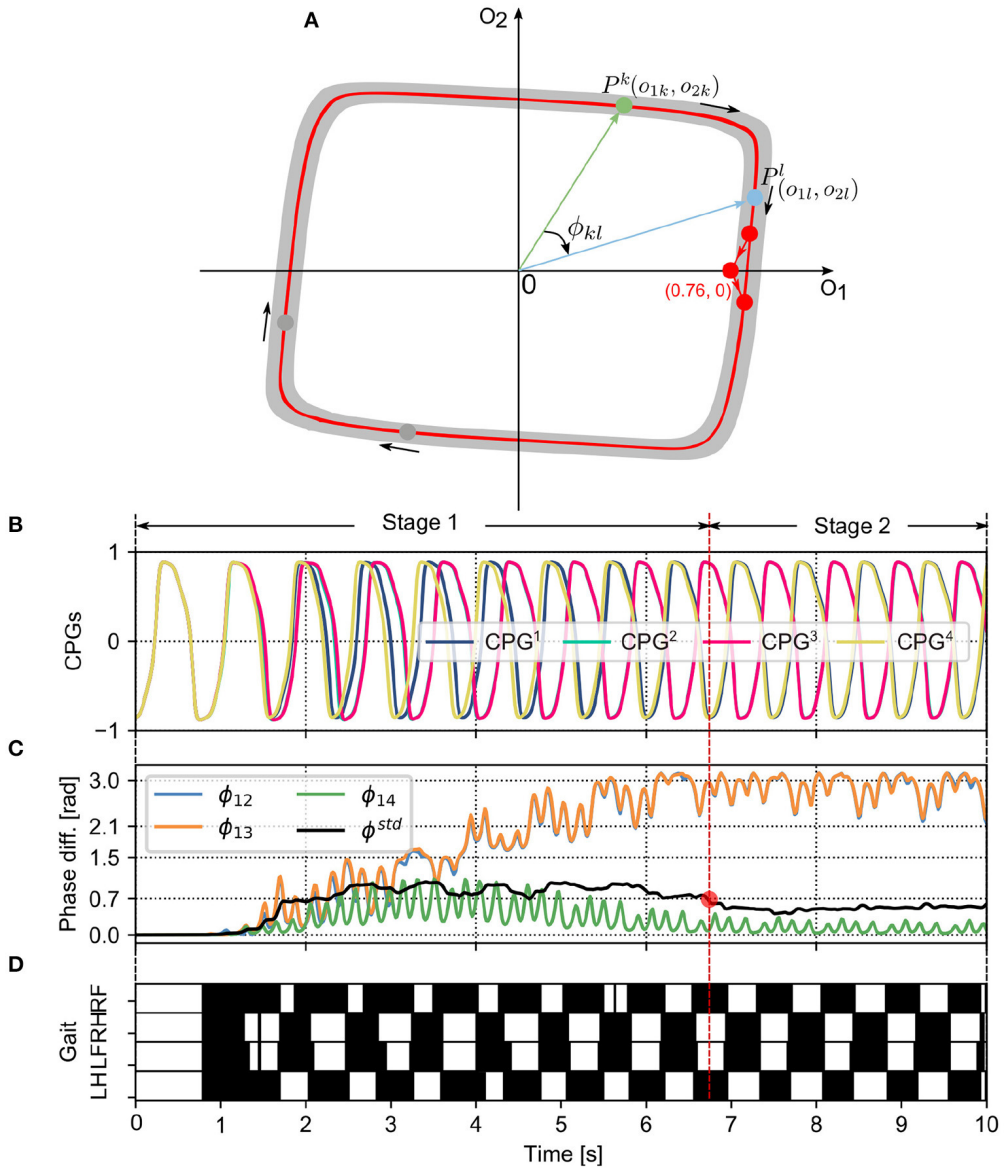
**(A)** The limit cycle of the SO(2)-based CPGs that was used to investigate the autonomous phase regulation. The coordinates (0.76, 0) represent the phase-reset point realized by the PR. A phase difference (e.g., ϕ_*kl*_) between two CPGs (i.e., the *k*th and *l*th CPGs) is defined as the angle between the two points (i.e., *P*^*k*^ and *P*^*l*^). **(B)** The first neuron outputs (*o*_1*k*_ with k=1, 2, 3, and 4) of the four CPGs that are used to control the four legs, respectively (see Figure 1). **(C)** The CPG phase differences (i.e., ϕ_12_, ϕ_13_, ϕ_14_) and their standard deviation (ϕ^*std*^). ϕ^*std*^ can indirectly reflect the phase deviation. Empirically, once the value of ϕ^*std*^ reduces to < 0.7 (see the red point), the CPG outputs and phase differences become more stable. The CPG phase convergence process can be divided into two stages (Stage 1 and Stage 2) determined by the point. **(D)** In the corresponding gait diagram, the black areas indicate stance phases while the white areas indicate swing phases. Note that, ϕ_12_, ϕ_13_, and ϕ_14_ are the phase differences of the *CPG*^2^, *CPG*^3^, and *CPG*^4^ with respect to the *CPG*^1^, respectively. RF, RH, LF, and LH are the right front, right hind, left front, and left hind legs, respectively.

In this study, we focused on the autonomous phase regulation of decoupled CPGs modulated by the PM and PR, resulting in quadruped self-organized locomotion. Here, we consider a neural SO(2)-based CPG with specific dynamical properties in which the CPG with a certain frequency exhibits a limit cycle similar to a unit circle in phase space, as shown in Figure 3A. In other words, the PM and PR are used to modulate the CPG phase rather than adapting to other properties (for example, amplitudes, offsets, and frequency). As a result, under the CPG parameter setup in Equations (4) and (5), the phase relationship of the decoupled CPGs converges to a certain state where the quadruped robot can form a specific gait (i.e., trot-like gait).

To clearly analyze and assess the characteristics of the PM and PR for the CPG phase regulation, several variables and metrics (see Table 1) were introduced to measure their CPG phase convergence process and resulting self-organized locomotion (see Figure 3). The metrics were used to assess the PM and PR in the experiments. Because the variables are the basis of the metric definitions, the variables are here introduced in the following subsection first. They include the phase difference and its mean and standard deviation.

**Table 1:** List of the variables and defined metrics.

**Variables**	**Symbols**	**Metrics**	**Symbols**
Phase difference	ϕ_*kl*_(*n*)	Phase convergence time	*T*
Mean of phase difference	$\phi_{kl}^{mean}\left(n\right)$	phase deviation	ϕ^*s*^
Standard deviation of phase difference	$\phi_{kl}^{std}\left(n\right)$	Cost of transport	*COT*
Sum of standard deviation of phase differences	ϕ^*std*^(*n*)		

#### 2.3.1. Variables

A phase difference between two CPGs can identify the phase relationship of the two CPGs as well as the movement relationship between the two limbs/legs controlled by the two CPGs. The outputs of a CPG (e.g., *o*_*k*1_ and *o*_*k*2_) at a moment can be illustrated as a point (*P*^*k*^) in a phase diagram (see Figure 3A). The two axes of the phase diagram represent the CPG outputs *o*_1,2_. When the CPGs produce periodic signals (see Figure 3B), their outputs follow their limit cycle to move. The limit cycle of a neural SO (2)-based CPG is similar to a circle whose origin is at the center of the coordinate. In the adaptive neural controller, the four neural SO (2)-based CPGs are identical with the same parameter values, so their limit cycles are the same in the phase diagram. Therefore, a phase difference (e.g., ϕ_*kl*_) between two CPGs (i.e., the *k*th and *l*th CPGs) can be represented by the angle between the two points (i.e., *P*^*k*^ and *P*^*l*^). Its mathematical description is as follows:

(11) $$\begin{array}{r} \phi_{kl}=\arccos\left(\frac{P_{k}\cdot P_{l}}{\left||P_{k}||\;||P_{l}|\right|}\right), \end{array}$$

where ***P***_*k*_ and ***P***_*l*_ represent the vectors of the *k*th and *l*th CPGs in the phase diagram, respectively (Figure 3A). ϕ_*kl*_ ∈ [0, π] represents the magnitude of their (relative) phase difference. Based on this definition (ϕ_*kl*_), when the adaptive neural controller is implemented on the quadruped robot to generate self-organized locomotion (Figure 3D), one can find the phase differences (i.e., ϕ_12_ and ϕ_13_) change from in phase to stable phase relationships (Figure 3B). As a result, the phase differences among the CPGs can display their phase relationship online (see Figure 3C). A video to show the phase difference convergences of the four decoupled CPGs modulated by the PM and PR can be seen in http://www.manoonpong.com/AICM/video1.mp4.

The phase differences undulate during the phase convergence process. To monitor the undulation, the mean and standard deviation of the phase differences are introduced. Because ϕ_*kl*_ ∈ [0,π] changes in a linear manner, it can be regarded as linear data rather than circular data when calculating its statistical variables. Thus, the mean and standard deviation are described as follows:

(12) $$\phi_{kl}^{mean}(n)=\left\{ \begin{array}{l} \frac{1}{N}\sum_{i=n-N}^{n}\phi_{kl}(i),\;n>N\\ \;\frac{1}{n}\sum_{i=0}^{n}\phi_{kl}(i),\;n\leq N\; \end{array}\right.,$$

(13) $$\phi_{kl}^{std}(n)=\left\{ \begin{array}{l} \frac{1}{N}\sqrt{\sum_{i=n-N}^{n}{\left(\phi_{kl}(i)-\phi_{kl}^{mean}(n)\right)}^{2},}\;n>N\\ \;\frac{1}{n}\sqrt{\sum_{i=1}^{n}{\left(\phi_{kl}(i)-\phi_{kl}^{mean}(n)\right)}^{2},}\;n\leq N\; \end{array}\right.,$$

(14) $$\begin{array}{r} \phi^{std}\left(n\right)=\overset{4}{\underset{l=2}{\sum }}\phi_{1l}^{std}\left(n\right), \end{array}$$

where $\phi_{kl}^{mean}\left(n\right)$ and $\phi_{kl}^{std}\left(n\right)$ are the mean and standard deviation of the phase difference ϕ_*kl*_ at current step *n*, respectively. *N* is the number of steps in a period from the current to a previous step. It is empirically set to 50 in the following experiments. Here, ϕ^*std*^(*n*) is the sum of $\phi_{12}^{std}\left(n\right),\phi_{13}^{std}\left(n\right),\text{and}\phi_{14}^{std}\left(n\right)$ at the *n*th step. This can reflect the instantaneous/current deviation of the phase differences in overall. The less ϕ^*std*^(*n*), the higher the phase deviation at the *n*th step.

To identify whether the CPG phase relationships are so stable that self-organized locomotion is recognized to be formed, according to the instantaneous indication of the phase deviation [ϕ^*std*^(*n*)], a constant $\phi_{t}^{std}$ is introduced as a threshold for distinguishing the phase convergence process. It is empirically set to 0.7 in the following experiments.

#### 2.3.2. Metrics

Based on the proposed variables (see Table 1), the first metric is phase convergence time, which indicates how long the CPG phase relationship takes to converge and the robot takes to generate self-organized locomotion under the restrict conditions. The state transition of the decoupled CPGs with the PM/PR from the initial fixpoint (0, 0, 0) to the desired fixpoint (π, π, 0) is accompanied by a process in which ϕ^*std*^ first increases and then decreases. Based on many experiments, we realize that if ϕ^*std*^ first reduces to less than a threshold ($\phi_{t}^{std}$ = 0.75) from a high value, the dynamical system will converge, and the quadruped robot can form a trot-like gait. Thus, the phase convergence time (*T*) is described as:

(15) $$\begin{array}{r} T=\frac{\min\left(n_{i}\right)}{H},\;\phi^{std}\left(n_{i}-1\right)\geq \phi_{t}^{std},\phi^{std}\left(n_{i}\right)<\phi_{t}^{std}, \end{array}$$

where $\phi_{t}^{std}$ is the threshold. *n*_*i*_ is the step when ϕ^*std*^ is reduced to less than $\phi_{t}^{std}$ in a trial, whereas min(*n*_*i*_) is the minimal value of *n*_*i*_ and represents the step when ϕ^*std*^ first reduces to less than the threshold. *H* is the update frequency of the control node (i.e., 60 Hz).

The second metric is *phase deviation*, which estimates the deviation of the phase differences. It can reflect the extent to which the converged CPG phase relationships are sustained during a self-organized locomotion period. It is defined using the reciprocal of the mean of ϕ^*std*^(*n*) as follows:

(16) $$\begin{array}{r} \phi^{s}=\frac{1}{mean\left(\phi^{std}\left(n\right)\right)},\;mean\left(\phi^{std}\left(n\right)\right)\neq 0, \end{array}$$

where *mean*(ϕ^*std*^(*n*)) represents the mean of ϕ^*std*^ in the period (e.g., with *M* steps). The greater ϕ^*s*^, the higher the phase deviation of the formed self-organized locomotion over the period.

The last metric is the *cost of transport (COT)*. It is used to measure the energy efficiency of the formed self-organized locomotion over a period. The COT is described as bellows:

(17) $$\left\{ \begin{array}{l} COT=\frac{E}{mgd},\\ E=\sum_{j=1}^{12}\sum_{n=1}^{M}\frac{I_{j}(n)V_{j}(n)}{H}, \end{array}\right.$$

where *E* is the energy consumption when the robot with weight *mg* travels with a distance *d*. The energy is calculated using the robot joint motor current *I*_*j*_(*n*) and voltage *V*_*j*_(*n*). *M* indicates the number of steps over the period. *H* is the update frequency of the experimental system.

## 3. Experimental Results

To systematically analyze and compare the characteristics of the PM and PR for self-organized locomotion, three robot experiments were conducted to measure the proposed metrics. First, the phase convergence time (see Equation 15) of the PM and PR under different parameter values was investigated. Subsequently, the phase convergence time of the PM and PR under different robot situations (i.e., a normal situation as a baseline, noisy feedback, leg damage, and carrying a load) were compared. Finally, the phase deviation (see Equation 16) and COT (see Equation 17) under the robot situations were also studied. More than 15 trials were conducted for each experiment under each mechanism (i.e., the PM or PR). Each trial was performed for more than 35 s.

At the beginning of each trial, an identical initialization procedure was conducted to maintain all experimental trials with the same initial conditions when the PM/PR was activated (initial state). The initialization required 270 time steps of 13.5 s, from the start of the simulation (*n* = 0) to the moment of dropping the robot on the ground (*n* = *n*_0_, where *n*_0_ = 270 in the following experiments). This initialization duration was selected to provide sufficient time to fulfill three settings: (1) setting/initializing the GRFs [*F*_*k*_(*n*_0_)] to zero by holding the robot in the air; (2) setting the joints of the four legs to the initial positions [θ_*ik*_(*n*_0_)] at the beginning of the simulation in all trials, so that the four legs had the same initial movement when the robot was dropped on the ground; (3) setting the CPG weights and biases to the initial values shown in Equations (4) and (5). The four neural SO(2)-based CPGs had the same parameter values and performed as the quasi-periodic attractors (see Figure 3A). As a result, the four CPGs generated stable periodic signals [*o*_*ik*_(*n*_0_)] in phase to control leg movement in the initial state (see Figure 3B).

### 3.1. Phase Convergence Time Under Different Parameter Values

**Figure 4:**
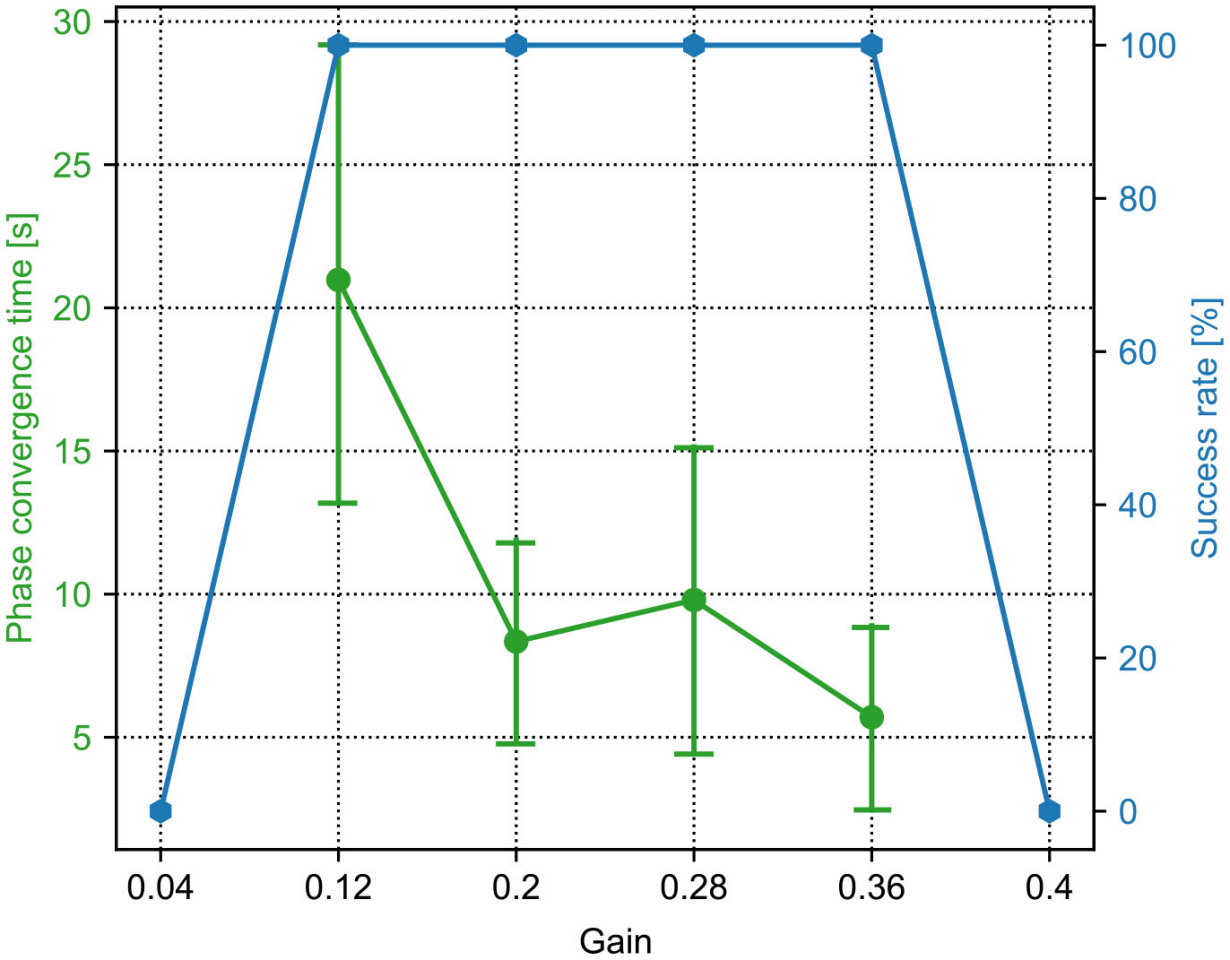
The phase convergence time and success rate of the PM trials with different sensory feedback gains (γ in Equation 6). The green points and bars show the average and variance of the phase convergence time, respectively. The blue points represent the success rate. When the gain is 0.36, the success rate is 100% and has the fastest phase convergence.

**Figure 5:**
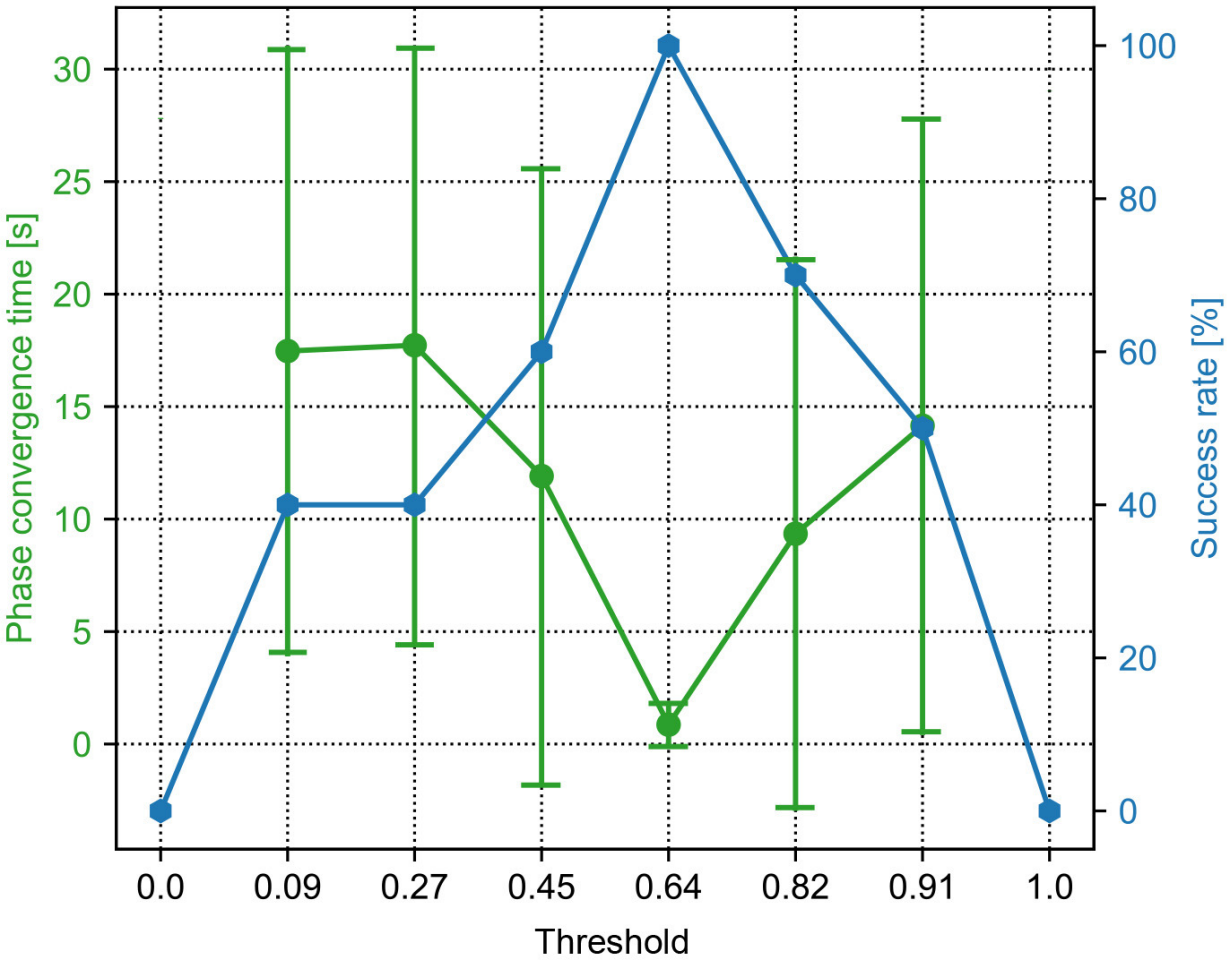
The phase convergence time and success rate of the PR trials with different force threshold factors (*F*_*t*_ in Equation 6). The green points and bars show the average and variance of the phase convergence time, respectively. The blue points represent the success rate. When the threshold factor is 0.64, the success rate is 100% and has the fastest phase convergence.

From Equations (6) and (8), it is known that the PM and PR parameters (i.e., sensory feedback gain γ and force threshold factor *F*_*t*_) play a key role in the CPG phase convergence. Therefore, this experiment investigated the optimal parameter values for fast CPG phase convergence through massive trails. To do that, the proposed adaptive neural controller with the PM or PR was applied to the robot. After initialization, the robot was placed on the ground, and it started to interact with the environment to form self-organized locomotion. The experimental results are depicted in Figures 4, 5.

For the PM, a sequence of the sensory feedback gains from 0.0 to 1.0 was tested. The range of the gain (i.e., 0.04, 0.12, 0.2, 0.28, 0.36, and 0.4) is shown in Figure 4. The other parameter values are not shown because they cannot enable the CPG phase differences to converge in all 15 trials. In the figure, the phase convergence time and success rate within 15 trials were recorded. Obviously, when the gain is in the range of [0.12, 0.36], the success rate is 100%. This means that the PM with these parameter values enables the robot to generate self-organized gait robustly in all 15 trials. One can also find that the best value of the gain is 0.36, by which the average phase convergence time is ~6 s. Consequently, the fastest phase convergence speed of the PM can be realized by setting γ to 0.36. This value was used for the PM in the following experiments.

For the PR, a sequence of the force threshold factor from 0.0 to 1.5 was tested. The range of the threshold (i.e., 0.0, 0.09, 0.27, 0.45, 0.64, 0.82, 0.91, and 1.0) is shown in Figure 5. The other parameter values are not shown because they cannot enable the CPG phase differences to converge in all 15 trials. In the figure, the phase convergence time and success rate within 15 trials were recorded. Obviously, when the threshold factor is in the range of [0.09, 0.91], the success rate is ≥40%. Especially, when the threshold factor is 0.64, the success rate is 100%. This means that the PR with the parameter value enables the robot to generate self-organized gait robustly in all 15 trials. In addition, the corresponding average phase convergence time is just approximately a second with a small derivation. Consequently, 0.64 is the optimal parameter value of the PR for the fastest phase convergence speed. This value was also used for the PR in the following experiments.

A success rate of 0 and 100% implies that the robot could not and could perform self-organized locomotion in all 15 trials. The basis for determining whether the robot forms self-organized locomotion (walking pattern) is that the phase differences (ϕ_12_, ϕ_13_, ϕ_14_) among the four CPGs converge to particular states around the desired fixpoint (π,π, 0) or the sum of their standard deviation (ϕ_*std*_) first reduces to less than a threshold (i.e., 0.7). For example, if the robot can perform a trot-like gait, the phase differences (ϕ_12_, ϕ_13_, ϕ_14_) should converge to approximately (π, π, 0) (see Supplementary Figures 1, 2).

### 3.2. Phase Convergence Time in Different Situations

**Figure 6:**
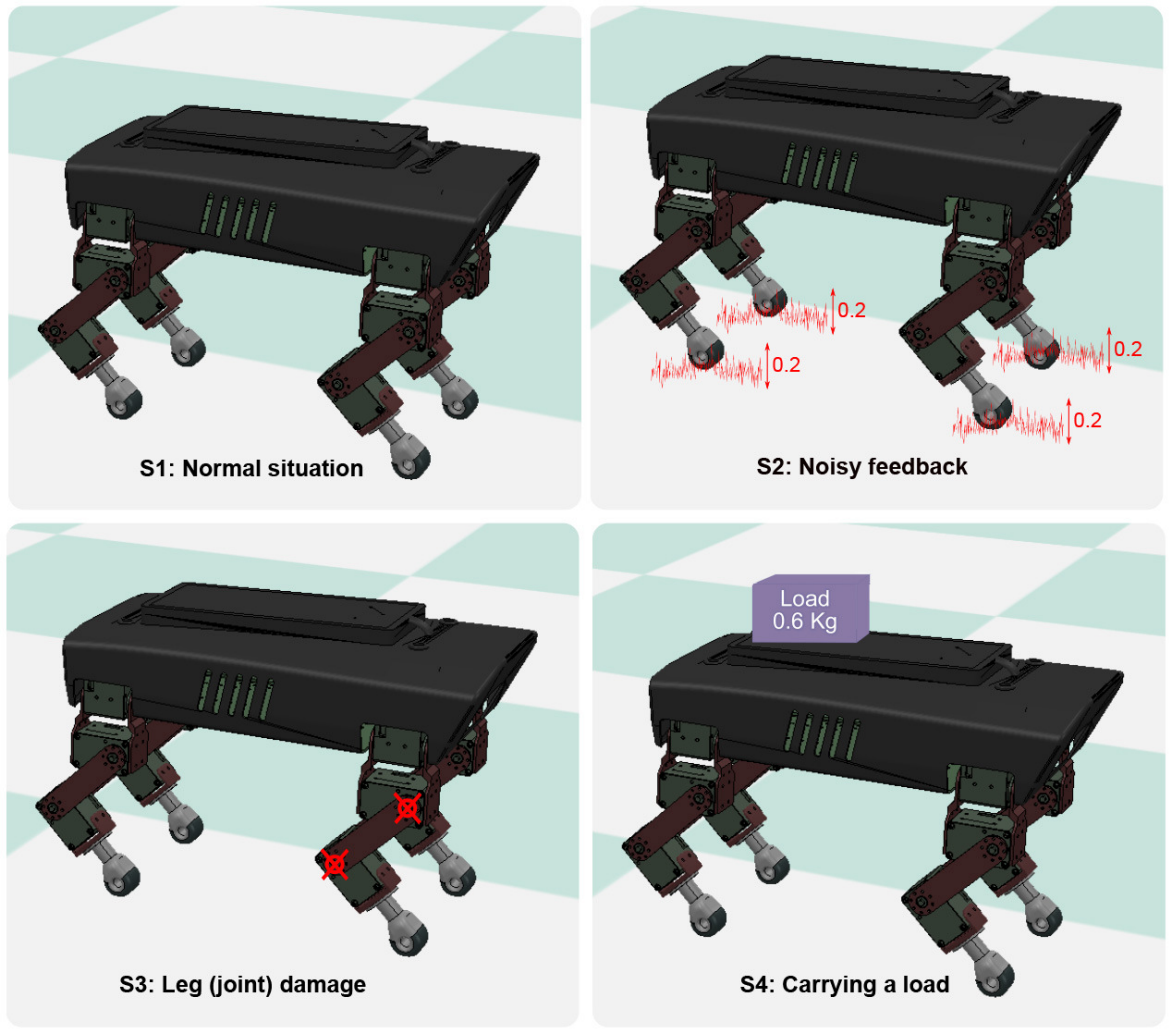
Four different situations that the robot experienced in the experiments. S1 was a normal situation. In S2, the GRFs of the four legs were added with Gaussian noise. In S3, the hip joint and knee joint of the right front leg were fixed to imitate leg damage. In S4, the robot carried a load of 0.6 kg.

The sensory feedback, GRF information, plays an essential role in the function of the PM and PR. To observe the adaptation of the PM and PR with respect to the GRFs, the PM and PR were examined in different robot situations, in which the robot might perceive different GRFs. The situations are illustrated in Figure 6. Their description can be seen in Table 2.

**Table 2:** The description of the four different situations that the robot experienced in the experiments.

**Situations**	**Description**
S1 (normal situation)	This was a normal situation. It served as a baseline for comparison with other unexpected situations.
S2 (noisy feedback)	The GRFs of four legs were added with Gaussian noise with an amplitude of 20% of the maximum value of the GRFs.
S3 (leg damage)	The hip and knee joints of the right front leg were fixed, so the right front leg was unable to move during the experiments.
S4 (carrying a load)	The experiment robot (Lilibot) carried a 0.6 kg load, and the load was near its hind legs.

The abnormal situations (S2, S3, and S4) were used to compare the functional properties of the PM and PR. The parameter settings of the abnormal situations were determined empirically to distinguish them from the normal situation (S1). In the S2 situation, Gaussian-distributed noise was empirically determined based on a trade-off between significant noise effects and the undisturbed phase regulation function of the PM and PR. Consequently, we used Gaussian distributed noise with a standard deviation of 20% of the GRFs. In the S4 situation, the weight of the payload was selected based on a trade-off between obviously distinct GRFs of the legs and the robot load capability.

**Figure 7:**
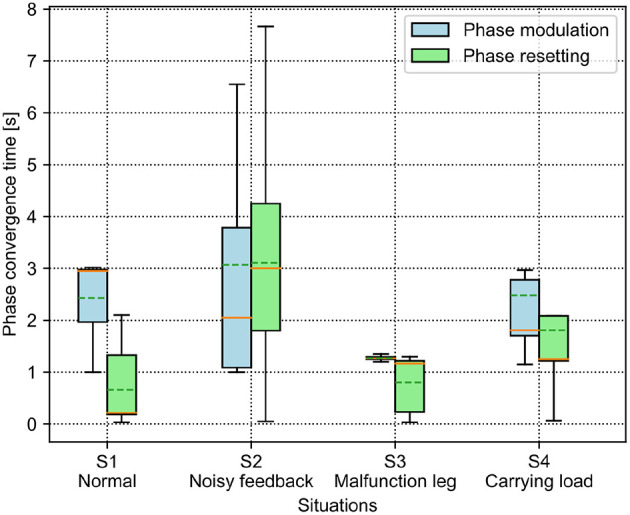
Phase convergence time of the PM and PR in four different situations. The solid and dashed lines in the boxes indicate the median and mean values of the phase convergence time, respectively.

The experiments were also performed by implementing the adaptive neural controller with the PM or PR on the quadruped robot but in the four situations. A video to show the robot generating self-organized locomotion under the PM and PR in the four situations are shown in http://www.manoonpong.com/AICM/video2.mp4. The experimental results can be seen in Figure 7.

For the PM, the average phase convergence time is <3 s in all situations. The best performance is in the S3 situation with the lowest average and variance of the phase convergence time, while the worst is in the S2 situation with the largest variance. Moreover, some trials in the S2 situation require more than 6 s to realize phase convergence. Overall, the unexpected situations (i.e., S2, S3, and S4) have faster phase convergence than that of the normal situation (S1). This is because the unexpected situations induced significant differentiation among the GRFs which can speed up the phase difference convergence.

For the PR, the phase convergence time of every situation in some trails is less than a second. Moreover, the average phase convergence time is <2 s, except for in the S2 situation, which exhibits the worst performance with the largest average and variance of the phase convergence time. Some trails cost more than 7 s to realize phase convergence in the S2 situation. This is because the added sensory noise made the GRFs randomly cross the force threshold so that the regular phase resetting process was destroyed. In the worst case, the CPG phase would never be reset.

To compare the results, the PR shows faster phase convergence than the PM on average, except for the trials in the S2 situation. This is because the PR rapidly reset the CPG phases once the GRFs increased over the threshold (i.e., 0.64) while the PM utilized the continuous GRFs with the gain (i.e., 0.36) to adjust the CPG phases smoothly. Consequently, the continuous phase modulation of the PM can cause slower but stable phase convergence. The rapid but intermittent phase resetting of the PR can cause faster phase convergence but with random success.

### 3.3. Phase Deviation and COT in Different Situations

After the CPG phase differences (ϕ_*kl*_) converge, the robot exhibits self-organized locomotion. It is also important to study how the phase differences and the formed locomotion are maintained. Therefore, this experiment exploited the deviation of the converged phase differences and used energy efficiency to assess the self-organized locomotion in the various situations.

**Figure 8:**
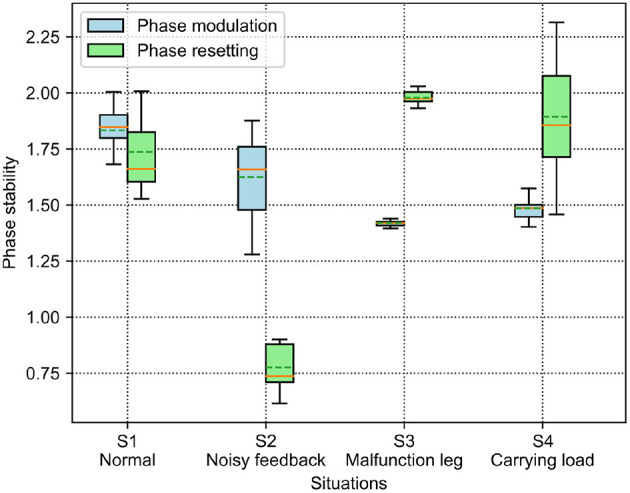
Phase deviation of the self-organized robot locomotion under the PM and PR in the four situations. The solid and dashed lines in the boxes indicate the median and mean values of the phase deviation, respectively.

The results of the phase deviation are shown in Figure 8. For the PM, the S1 situation has the greatest average phase deviation among the four situations. Specifically, the average phase deviation in the S1 and S2 situations is >1.5, while it is <1.5 in the other two situations. For the PR, the S2 situation has a large drop in the average phase deviation compared with the other situations. Specifically, the average phase deviation in the S1 and S2 situations is <1.75, while it is >1.75 in the other two situations. Comparatively, the average phase deviation of the PM is higher than that of the PR in the S1 and S2 situations, but lower than that of the PR in the S3 and S4 situations.

**Figure 9:**
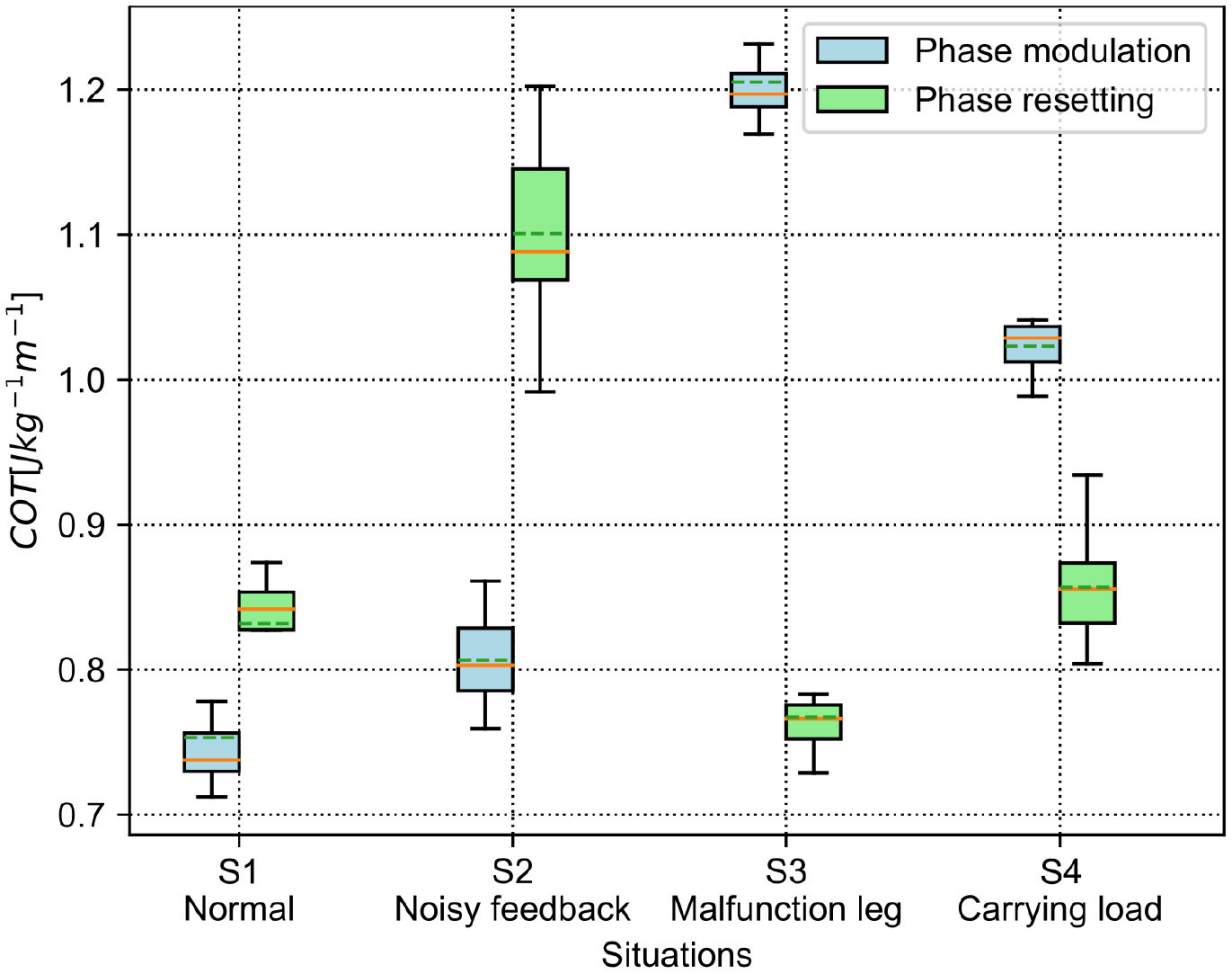
COT of the self-organized robot locomotion under the PM and PR in the four situations. The solid and dashed lines in the boxes indicate the median and mean values of the COT, respectively.

The results of the energy efficiency (measured by COT) are shown in Figure 9. For the PM, the lowest and the highest average COT are in the S1 and S3 situations, respectively. Specifically, the average COT in the S1 and S2 situations is <0.9, while it is >0.9 in the S3 and S4 situations. For the PR, the S2 situation has the highest COT in the four situations. Comparatively, the average COT of the PM is less than that of the PR in the S1 and S2 situations, but higher than that of the PR in the S3 and S4 situations.

According to the results shown in Figures 8, 9, the statistical analysis reveals that the PM has higher phase deviation and energy efficiency (lower COT value) than those of the PR in the S1 and S2 situations, while this result is reversed in the S3 and S4 situations.

**Figure 10:**
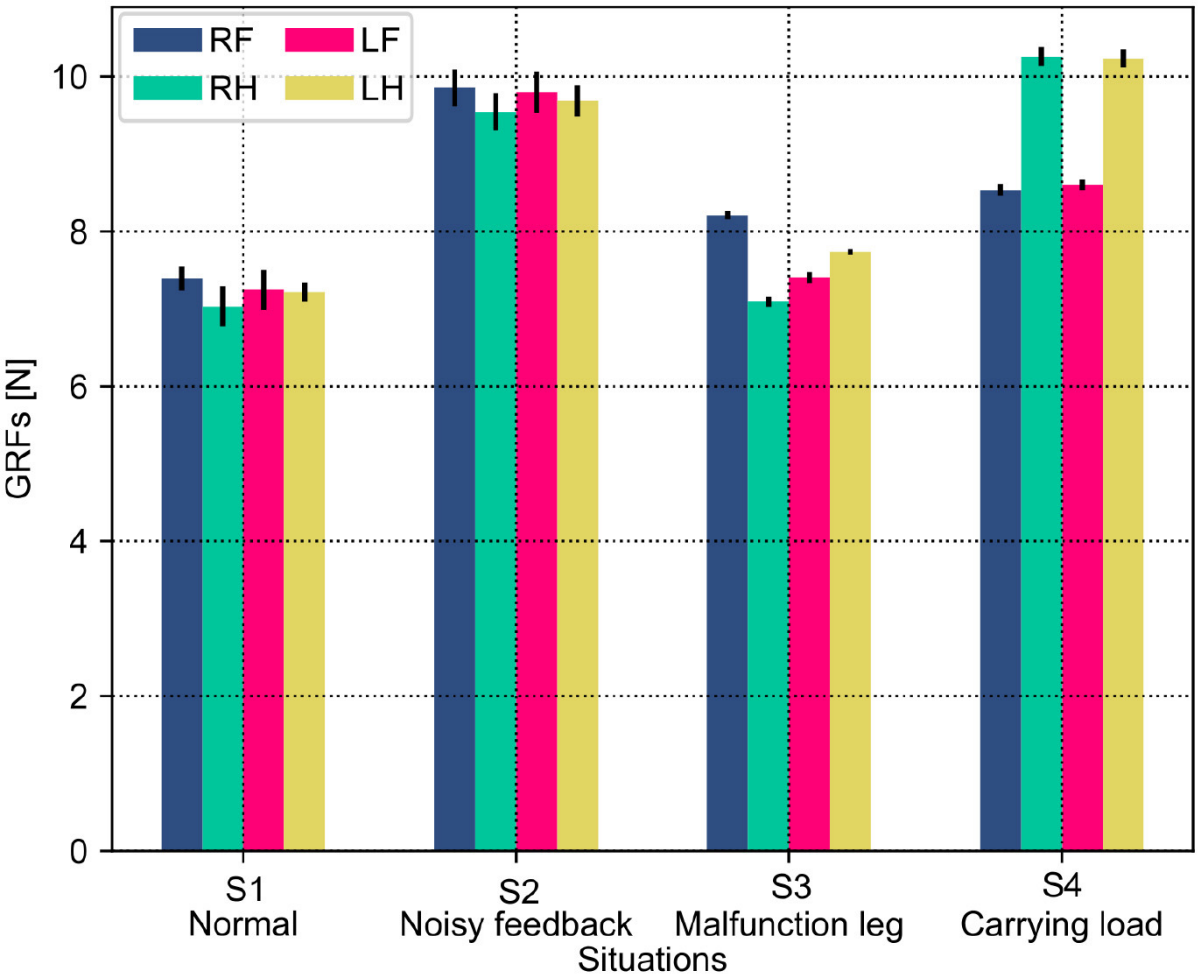
GRF distribution of the self-organized robot locomotion under the PM in four situations. Note that RF, RH, LF, and LH indicate the right front, right hind, left front, and left hind legs, respectively.

**Figure 11:**
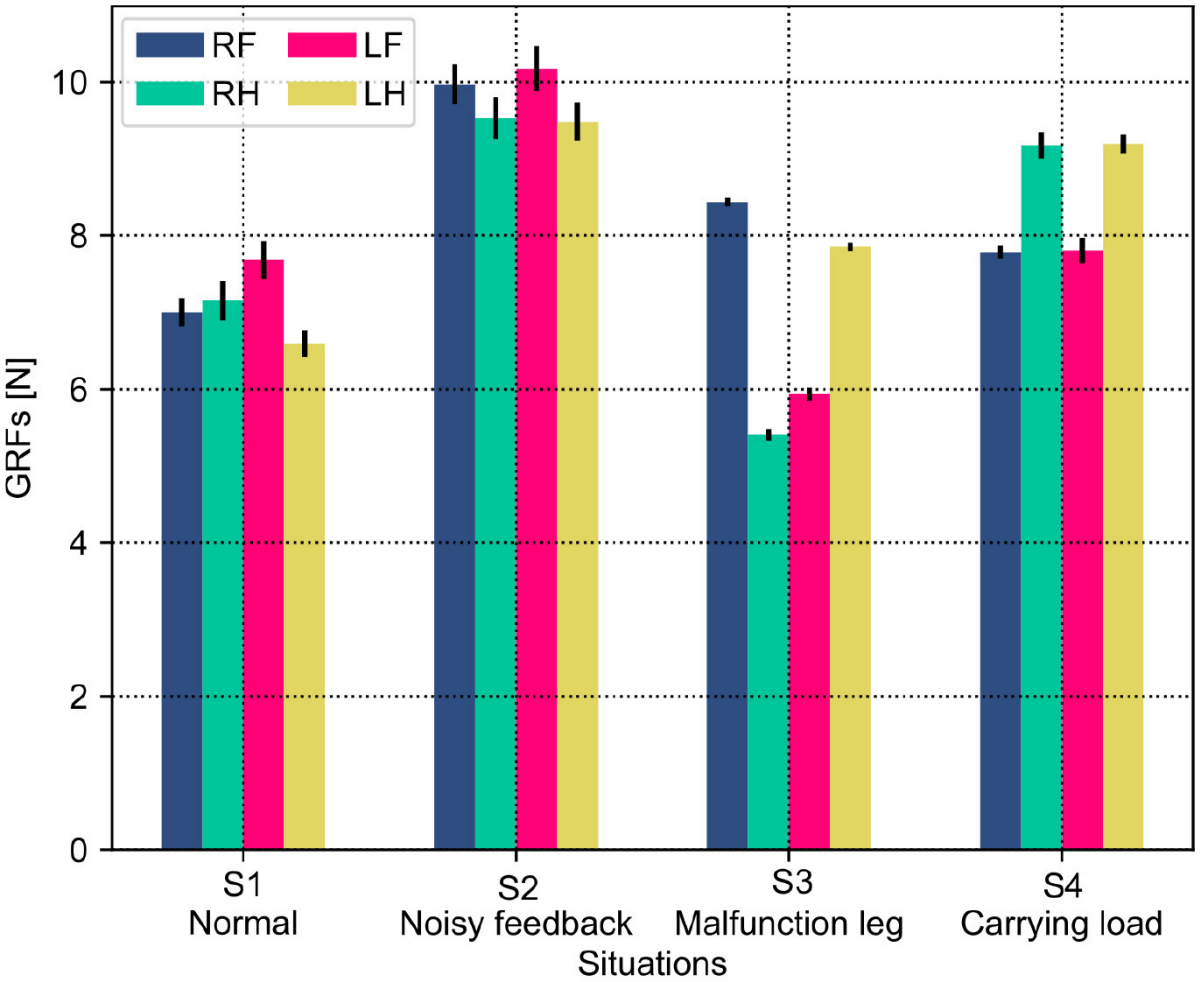
GRF distribution of the self-organized robot locomotion under the PR in the four situations. Note that RF, RH, LF, and LH indicate the right front, right hind, left front, and left hind legs, respectively.

Both the PM and PR have different performances (i.e., phase deviation and COT) in these situations. This results from the situations causing the robot to perceive different GRF distributions. The statistical GRFs under the PM and PR in the experiments are shown in Figures 10, 11, respectively.

In Figure 10, under the PM, the four legs (i.e., the RF, RH, LF, and LH legs) show more similar GRFs values in the S1 and S2 situations than in S3 and S4 situations. This phenomenon can also be seen in Figure 11 under the PR. The GRF distributions of the four legs in the S1 and S2 situations are symmetric, while, in the S3 and S4 situations, the GRFs show relative asymmetry. Taken together, the PM shows higher phase deviation and energy efficiency when facing a symmetric GRF distribution, while the PR shows higher performance when facing an asymmetric GRF distribution.

## 4. Discussion and Conclusion

The aim of this study was to comparatively analyze the characteristics of the two classical adaptive interlimb coordination mechanisms, the PM (see Equation 6) and PR (see Equation 7), for autonomous CPG phase regulation and resulting self-organized locomotion and adaptation. The essential functions of the PM and PR represent two different ways to regulate the phase relationships among decoupled CPGs. Typically, the PM uses continuous GRFs to modulate CPG phases gradually while the PR uses discrete GRFs to reset the CPG phases intermittently. In this study, the two mechanisms were separately applied to the adaptive neural controller with four decoupled SO (2)-based CPGs (see Figure 1). They were implemented on the quadruped robot to experimentally assess the PM's and PR's parameters and adaptability to unexpected robot situations (see Figure 6). The experimental results indicate that (1) the PM and PR parameter values significantly influence the success rate and speed of the CPG phase convergences; (2) overall, the PM exhibits slower but more stable phase convergence while the PR exhibits faster but less stable phase convergence (see Figures 4, 5); (3) the CPG phase convergence time varies in different situations (see Figure 7); and (4) the PM and PR perform better when the robot is subjected to symmetrical and asymmetrical GRF distributions, respectively (see Figures 8–11).

The decoupled CPGs with the PM/PR form a complex dynamical system that comprises three sublevels. Its difference equations can be seen in Equations (1), (6), and (7). (1) The top sublevel dynamical system comprises four identical and decoupled CPGs with the PM or PR, the state variables of which are the CPG phase differences (i.e., ϕ_12_, ϕ_13_, and ϕ_14_). (2) The middle sublevel dynamical system is a CPG with the PM or PR. The PM or PR term can be regarded as external adjustments on the CPG (basis sublevel dynamical system) when the robot interacts with the ground. (3) The basis sublevel dynamical system is a neural SO(2)-based CPG. Its state variables are the CPG outputs (*o*_*ik*_, i = 1, 2). Here, it is an oscillatory system under the proper parameter configuration (see Equations 4 and 5). Its dynamics is a limit cycle in the phase space (see Figure 3A). The initial conditions of a multiple-coupling CPG system strongly influence the convergence results (Dénes et al., 2019). In this work, the initial condition of the top sublevel dynamical system is the CPG coordination [*o*_1*k*_(*n*_0_), *o*_2*k*_(*n*_0_)] at the CPG limit cycle when the robot lands on the ground (*n* = *n*_0_). Thus, the ensemble of the initial conditions of the top dynamical system is the entire CPG limit cycle. In all experiments, we considered the initial condition of the time 270 steps (*n*_0_ = 270) where *o*_1*k*_(*n*_0_) ≈ 0.836 and *o*_2*k*_(*n*_0_) ≈ 0.067.

The convergence results (e.g., success rate) of the top sublevel dynamical system depend on the initial condition as well as the PM and PR parameter values [sensory feedback gain (γ) and GRF threshold (*F*_*t*_)]. When the PM and PR parameter values are outside their effective range (e.g., γ ∉ [0.12, 0.6] and *F*_*t*_ ∉ [0.09, 0.91], see Figures 4, 5), the robot cannot achieve self-organized locomotion (success rate is 0%) regardless of any initial condition. In this case, the top sublevel dynamical system always stays at an initial fixpoint (0,0,0) (see Supplementary Figures 1, 2). This is because the PM and PR with inappropriate parameter values cannot drive the system dynamics from the initial fixpoint to the desired fixpoint (π, π, 0) where a gait can be formed. More specifically, for the PM, if γ < 0.12 (e.g., γ = 0, Supplementary Figure 3), the sensory feedback strength is extremely weak to modulate the CPG phase; if γ > 0.6 (e.g., γ = 1, Supplementary Figure 5), the sensory feedback modulation is extremely strong, thereby significantly changing the CPG properties (e.g., output amplitudes and offsets). For the PR, if *F*_*t*_ < 0.09 (e.g., *F*_*t*_ = 0, Supplementary Figure 6), the four CPG phases are reset at the same time so that their phase differences are zero; if *F*_*t*_ > 0.91 (e.g., *F*_*t*_ = 1.5, Supplementary Figure 8), the four CPG phases never reset because the sensory feedback cannot meet the phase-resetting condition.

The statistical results (success rate) of the self-organized locomotion are related to the initial condition and parameter values. For the PM, if the parameter value (γ) is in the range of [0.12, 0.6], the PM-based control enables the robot to generate self-organized locomotion with a 100% success rate. The experimental real-time data of the case (e.g., γ = 0.36) are shown in Supplementary Figure 4. The dynamical system converges to the desired fixpoint (π, π, 0) in the phase space (see Supplementary Figure 1). For the PR, if the parameter value (*F*_*t*_) is in the range of [0.09, 0.91], the PR-based control enables the robot to generate self-organized locomotion (e.g., *F*_*t*_ = 0.64, Supplementary Figure 7) with some uncertainties. The dynamical system can converge to the desired fixpoint (π, π, 0) in the phase space (Supplementary Figure 2). A slight difference in the initial condition may cause distinct convergence results. For example, when *F*_*t*_ is 0.45, in one trial (Supplementary Figure 9), the robot can perform self-organized locomotion; in another trial using the same parameter value and the same initial procedure, the robot cannot generate self-organized locomotion (see Supplementary Figure 10). This is because, in the success case, the GRFs of the four legs can cross the GRF threshold at slightly different times owing to slightly different dynamics among the four legs at the touch moment, even when the four legs touch the ground at the same time. This is because the GRFs of the four legs approached the GRF threshold with a slightly different increase rate when the robot touched the ground (see Supplementary Figure 9). According to this, the results based on the PR are more sensitive to the initial condition than those based on the PM.

The cases with a 0% success rate in Figures 4, 5 result from the inappropriate “physical coupling strength” of the CPGs. In this work, the adaptive synchronizations/coordination among the decoupled CPGs is realized via sensory feedback in the form of the PM or PR, which provides physical communication/coupling effects on the CPGs. The PM and PR parameter values (γ of the PM and *F*_*t*_ of the PR) determine the “physical coupling strength.” When the parameter values are extremely small or large, the “physical coupling strength” also becomes extremely small or large such that synchronization will not be achieved. As a result, the CPG phase relationships (ϕ_12_, ϕ_13_, and ϕ_14_) of the decoupled CPGs are not appropriate for forming a stable gait.

The PM and PR have been analyzed from various aspects in different ways in other works (Aoi et al., 2012; Owaki et al., 2013, 2017; Ambe et al., 2018). For instance, Owaki et al. (2013) have summarized the spontaneous phase shift of the decoupled CPGs, which are regulated by local force feedback in the form of the PM, as follows: (i) a phase delay is introduced in the CPG of each leg owing to the physical effect of the local force feedback; (ii) this phase delay, which is introduced when the leg is in a stance phase, allows time for other legs to enter the stance phase; (iii) as more legs begin to support the body, the load on the support leg decreases; consequently, the feedback effect on the support leg decreases, allowing it to enter the swing phase. The mechanism reveals how the phases of the CPG are appropriately modified by local sensory feedback, resulting in the generation of the self-organized locomotion. Ambe et al. (2018) analyzed the phase evolution of (no direct interaction) ipsilateral oscillators, which are regulated by local force feedback in the form of the phase resetting. In this case, the CPG phases are shifted and converge to the final state when the legs touch the ground at proper moment. This is because the force feedback can regulate the leg retraction timings by resetting the CPG phase.

However, in the above-mentioned studies the characteristics of the PM and PR models' parameters seem to receive less attention and have not been reported in detail. In this work, the effects of the parameters of the PM and PR on the CPG phase convergences were systematically investigated. As a result, their optimal normalized parameter values were found (see Figures 4, 5). This increases the practicality of the two mechanisms for obtaining fast phase convergence in the normal situation (i.e., the S1 situation) by reducing the manual parameter tuning. However, the phase convergence times vary in different robot situations (see Figure 7). This suggests that adaptive parameter values of the PM and PR are necessary in various situations. Recently, some studies have implemented learning techniques to obtain adaptive sensory feedback gains of the PM mechanisms (Sun et al., 2018; Dujany et al., 2020; Miguel-Blanco and Manoonpong, 2020).

Another important property of the PM and PR is their adaptability to changes in body properties. It has been reported in many works (Owaki et al., 2013, 2017; Ambe et al., 2018), in which researchers have reproduced certain impressive animal-like movements on legged robots, such as self-organized gaits and autonomous gait transition in response to changes in body properties (e.g., leg amputations and weight redistribution) and environments. These works viewed the adaptability in terms of adaptive walking patterns. In this work, the phase deviation (Equation 16) and energy efficiency (i.e., COT, see Equation 17) were exploited in four elaborated robot situations (see Figure 6).

The four situations varied the four legs' GRF amplitudes and exhibited two different GRF distributions: symmetrical GRFs (in the S1 and S2 situations) and asymmetrical GRFs (in the S3 and S4 situations). The experimental results show that the higher phase deviation of the CPGs corresponds to the higher energy efficiency of the self-organized locomotion. This reflects a straightforward relationship of the control metric to locomotion performance. The relationship maybe attributed to the higher phase deviation with fewer unpredictable joint movement changes, thereby saving energy cost. Moreover, the PM and PR exhibited good performance when they were subjected to symmetric and asymmetric GRF distributions, respectively. This indicates that the two mechanisms should be selected in different situations in the self-organized robot locomotion.

Taken together, the comparative study of the PM and PR in this work reveals not only the relationship between their parameter values and the speed of the self-organized locomotion generation, but also the preferred situations for high phase deviation and energy efficiency in locomotion. Based on this study, it suggests that the PM and PR are effective in different situations. However, these conclusions are based on the robot experiments with the specific neural SO(2)-based CPG setup and the simulated quadruped robot platform. This limits the generality of the conclusions in general CPG and legged robots. In addition, the definition of the phase convergence time depends on empirically tuned parameters (i.e., $\phi_{t}^{std}$ in Equation 15 and *N* in Equation 12), which were determined by observing the experiments implemented in our specific robotic platform. As a result, the statistical results of the phase convergence time, phase deviation (Figures 4, 5, 7, 8) could be affected by the experimental platform. Moreover, the metric ϕ^*std*^ is not monotonic and could crossover the threshold more than once, for example, in the S2 situation where the GRFs have additional noise (see Supplementary Figure 16). Thus, to obtain the same experimental conclusion on other experimental platforms, the empirical parameters should be adjusted manually according to a specific experimental platform. Thus, in future work, we will further theoretically investigate the two mechanisms based on a dynamical system perspective (Sándor et al., 2015; Aguilar et al., 2016; Martin et al., 2016; Dénes et al., 2019) to further analyze the properties of the mechanisms (e.g., using Poincaré map Owaki and Ishiguro, 2017) and structural stability and to verify the experimental results on other robotic systems, such as hexapod robots.

## Data Availability Statement

The raw data supporting the conclusions of this article will be made available by the authors, without undue reservation.

## Author Contributions

TS implemented the control methods, analyzed the data, and wrote the original manuscript. XX guided the experimental design and the research direction, as well as reviewed the manuscript. ZD supervised the study. DO supervised the study and reviewed the manuscript. PM fully supervised this study (including the research idea, experimental design, experimental data analysis) and wrote the manuscript. All authors contributed to the article and approved the submitted version.

## Conflict of Interest

The authors declare that the research was conducted in the absence of any commercial or financial relationships that could be construed as a potential conflict of interest.

## Footnotes

^1^Note that an SO (2)-based CPG is a special type of 2-neuron network where the weight matrix of the network is an element in the special orthogonal group SO(2).

^2^https://www.coppeliarobotics.com/

^3^https://www.cm-labs.com/vortex-studio/

^4^https://www.ros.org/
