# Corrigendum: [A Comparative Study of Adaptive Interlimb Coordination Mechanisms for Self-Organized Robot Locomotion]

**DOI:** 10.3389/frobt.2021.702167

**Published:** 2021-06-02

**Authors:** Tao Sun, Xiaofeng Xiong, Zhendong Dai, Dai Owaki, Poramate Manoonpong

**Affiliations:** ^1^ Institute of Bioinspired Structure and Surface Engineering, College of Mechanical and Electrical Engineering, Nanjing University of Aeronautics and Astronautics, Nanjing, China; ^2^ Embodied Artificial Intelligence and Neurorobotics Laboratory, SDU Biorobotics, The Mærsk Mc-Kinney Møller Institute, University of Southern Denmark, Odense, Denmark; ^3^ Department of Robotics, Graduate School of Engineering, Tohoku University, Sendai, Japan

**Keywords:** adaptive interlimb coordination, phase resetting, phase modulation, decoupled CPGs, sensory feedback, self-organized locomotion

In the original article, there were errors.

In the published article, there was an error in affiliation **1**. Instead of “**College of Mechanical and Electrical Engineering, Institute of Bio-inspired Structure and Surface Engineering, Nanjing University of Aeronautics and Astronautics, Nanjing, China**”, it should be “**Institute of Bio-inspired Structure and Surface Engineering, College of Mechanical and Electrical Engineering, Nanjing University of Aeronautics and Astronautics, Nanjing, China**”.

In the published article, there was an error in affiliation **2**. Instead of “**Embodied Artificial Intelligence and Neurobotics Lab, University of Southern Denmark Biorobotics, M**æ**rsk Mc-Kinney M**ø**ller Institute, University of Southern Denmark, Odense, Denmark**”, it should be “**Embodied Artificial Intelligence and Neurorobotics Laboratory, SDU Biorobotics, The M**æ**rsk Mc-Kinney M**ø**ller Institute, University of Southern Denmark, Odense, Denmark**”.

The authors apologize for this error and state that this does not change the scientific conclusions of the article in any way. The original article has been updated.

