# Supplementary material for: A Comparative Study of Adaptive Interlimb Coordination Mechanisms for Self-Organized Robot Locomotion
Source: Front Robot AI. 2021 Apr 12;8:638684. doi: 10.3389/frobt.2021.638684 (PMC8072274; doi:10.3389/frobt.2021.638684)
Supplement: Supplementary file 2 [file Data_Sheet_2.PDF]

Supplementary Material

1 ILLUSTRATIONS OF EXPERIMENTAL DATA

1.1 Experimental data under different parameter values

This subsection illustrates dynamical properties of the decoupled CPGs with the PM/PR (top level dynamical system), which is used to control a quadruped robot for generating self-organized locomotion automatically, under three different parameter values as examples. The three parameter values are the minimal, middle, and maximal parameter values of the PM/PR that we tested in the experiments, respectively. The PM parameter γ is set to 0.0, 0.36, and 1.0 in three trials, respectively (Figure S1). The PR parameter F_t is set to 0.0, 0.64, and 1.5 in three trials, respectively (Figure S2). In all trials under the PM and PR, the state variables (ϕ_{12}, ϕ_{13}, and ϕ_{14}) are set to the point (0,0,0) in the phase space through the experimental initialization procedure. One can find that the state variables automatically gather around a fix point (π, π, 0) when $\gamma = 0.36$ or $F_t = 0.64$ which corresponds to the robot walking with a trot-like gait.

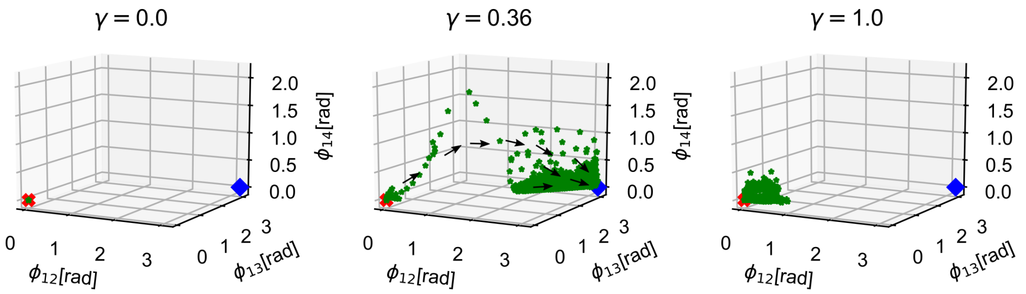

Figure S1. Phase portrait of the decoupled CPGs with the PM. The PM parameter γ is set to 0.0, 0.36, and 1.0 in three trials, respectively. When γ value is out its effective range (e.g., $\gamma = 0$ and $\gamma = 1$), the state variables of the dynamical system (represented by the green points) can not converge to the desired fixed points (i.e., (π, π, 0) blue point) from the initial fixed point ((0, 0, 0) red point). In contrast, the dynamical system can converge to around the desired fixed points when γ is in its effective range (e.g., $\gamma = 0.36$). In the case, the robot performs a trot-like gait.

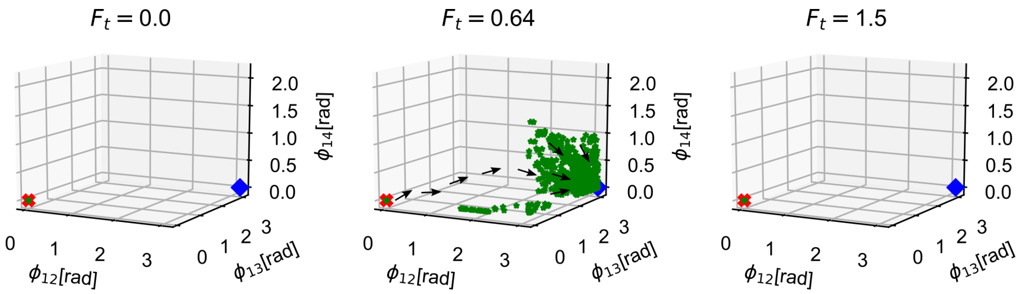

Figure S2. Phase portrait of the decoupled CPGs with the PR. The PR parameter F_t is set to 0.0, 0.64, and 1.0 in three trials, respectively. When F_t value is out its effective range (e.g., $F_t = 0$ and $F_t = 1.5$), the state variables of the dynamical system (represented by the green points) can not converge to around the desired fixed points (i.e., (π, π, 0) blue point) from the initial fixed point ((0, 0, 0) red point). In contrast, the dynamical system can converge to around the desired fixed point when F_t is in its effective range (e.g., $F_t = 0.64$). In the case, the robot performs a tort-like gait.

The real-time data of the above six trials are depicted in Figures S3, S4, S5, S6, S7, and S8, respectively. Each of these figure has five subplots, including 1) the first neuron output ($o_{1k}, k = 1, 2, 3, 4$) of the four neural SO(2)-based CPGs, 2) the CPG phase differences (ϕ_{12}, ϕ_{13}, and ϕ_{14}, or called state variables of the decoupled CPGs) and the sum standard deviation of the phase differences (ϕ^{std}), 3) the right-side legs' GRFs, 4) the left-side legs' GRFs, and 5) gait diagram. In gait diagram, white and black areas represent stance and swing phases, respectively. Note that, the RF, RH, LF, and LH indicates the right front, right hind, left front, and left hind legs in the figure, respectively.

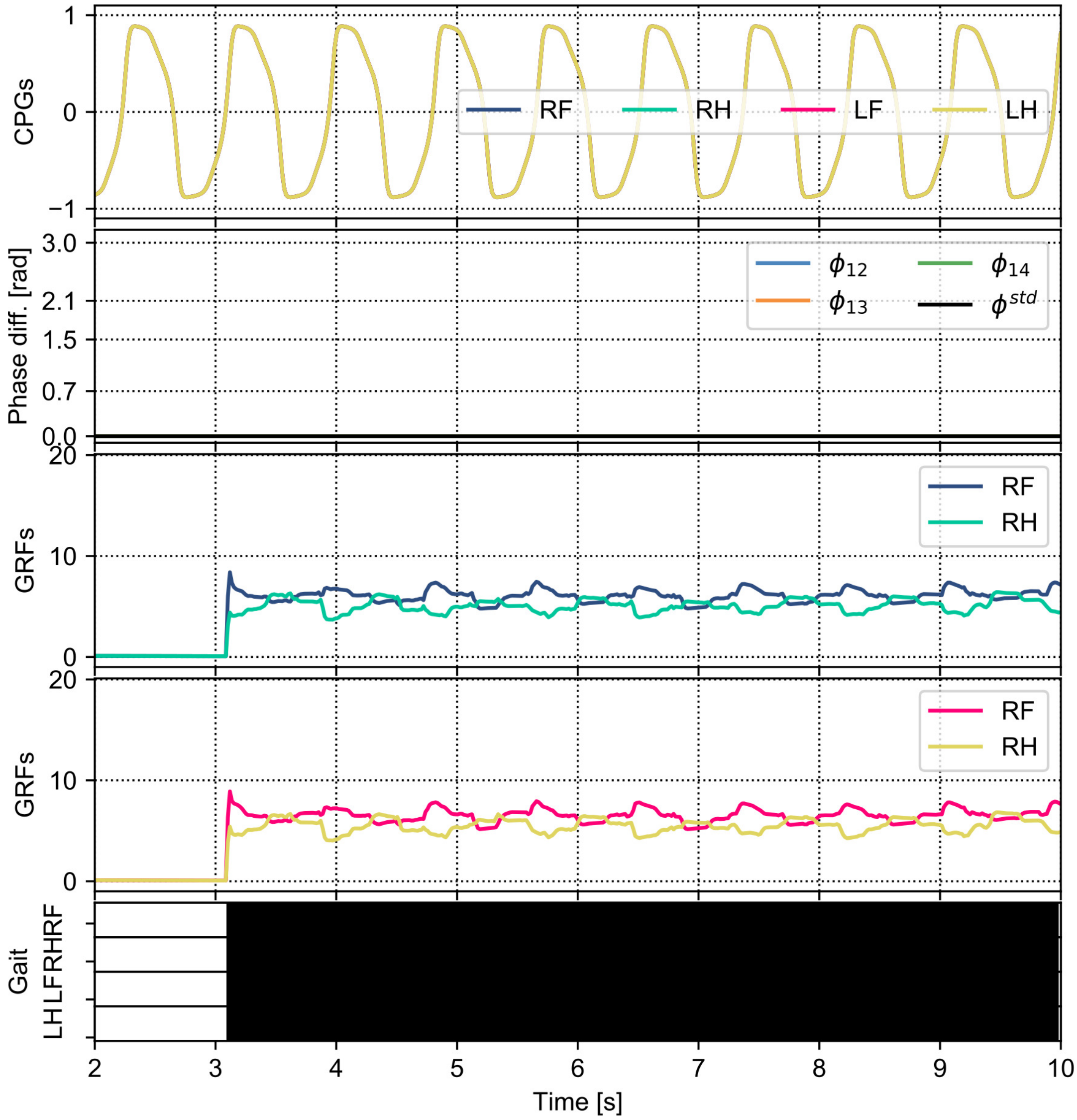

Figure S3. Experimental data of a trial under the PM with $\gamma = 0.0$. The sensory feedback to CPGs was not activated due to the sensory feedback gain is zero, thus no phase shifts occurred among the four CPGs. The phase differences were zeros, the robot four legs always stayed at stance phase without stepping forward.

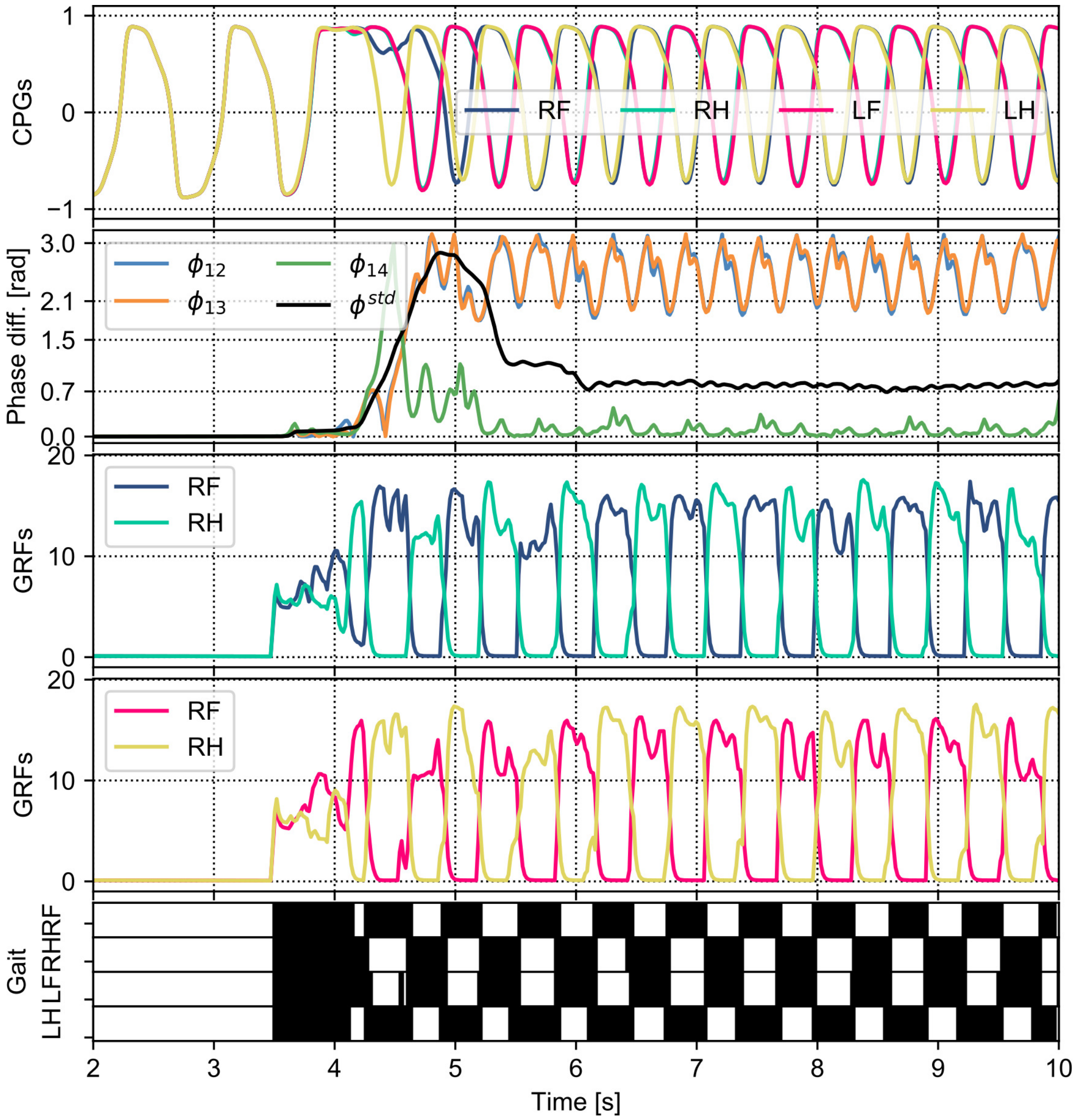

Figure S4. Experimental data of a trail under the PM with $\gamma = 0.36$. The sensory feedback with proper parameter value regulated the decoupled CPG phase, thus phase shifts occurred. The phase differences converged to around (π, π, 0), ϕ^{std} reduced to less than 0.75 after the self-organized robot locomotion emerged.

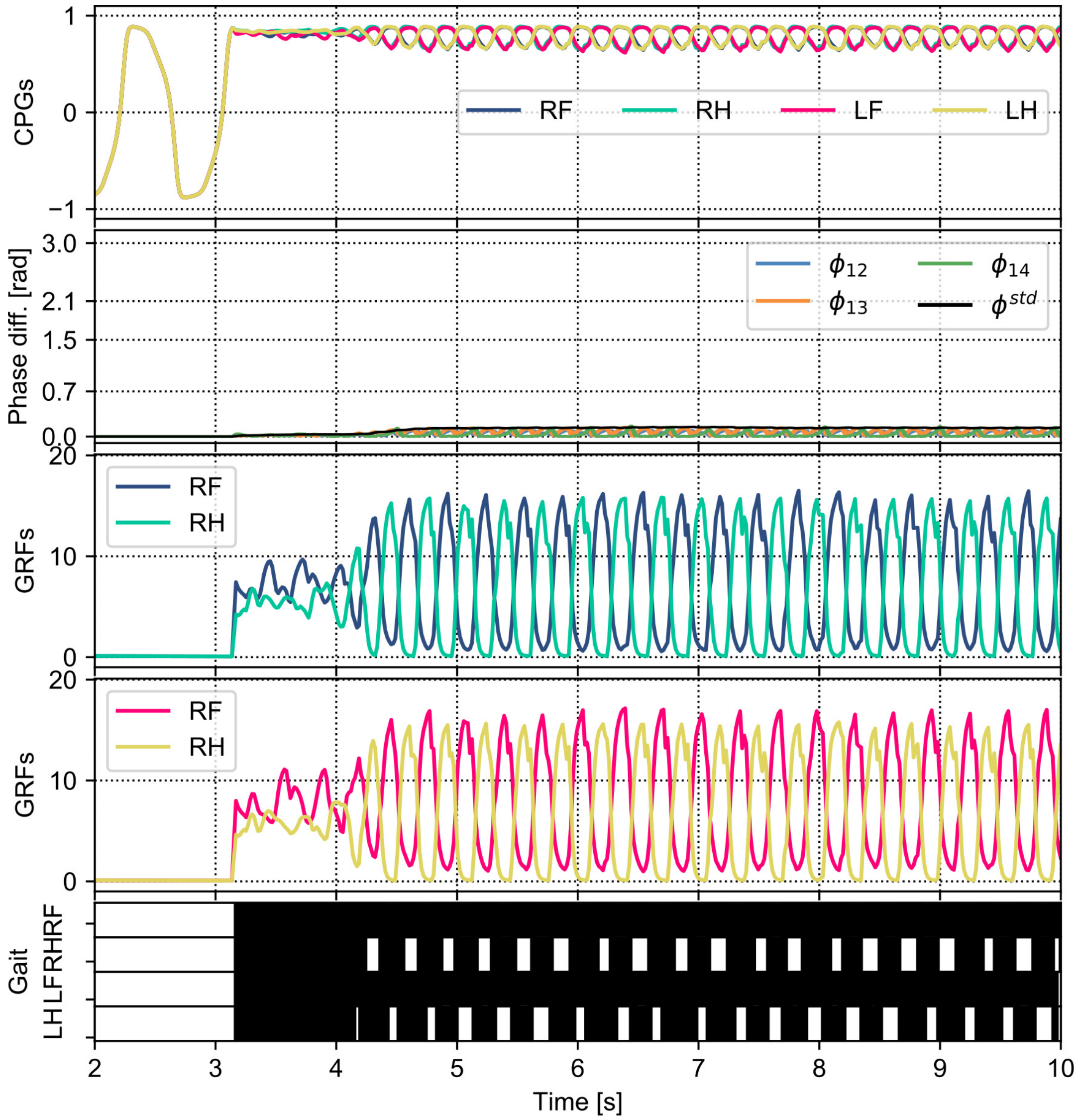

Figure S5. Experimental data of a trial under the PM with $\gamma = 1.0$. The sensory feedback with excessive gain inhibited the neural SO(2)-based CPG so much that the CPG outputs' amplitudes and offsets changed a lot. The CPGs' dynamical properties changed totally, its states were no longer in the restrict limit cycle (see Figure 3 (A) of the main manuscript.). In this work, we just compared the PM and PR based on the specific CPG dynamics, thus we did not counter this having a successful self-organized locomotion generation. Although the gait diagram shows the right hind and left hind legs with an alernative and short swing phase, the robot just struggled at the starting point on the ground.

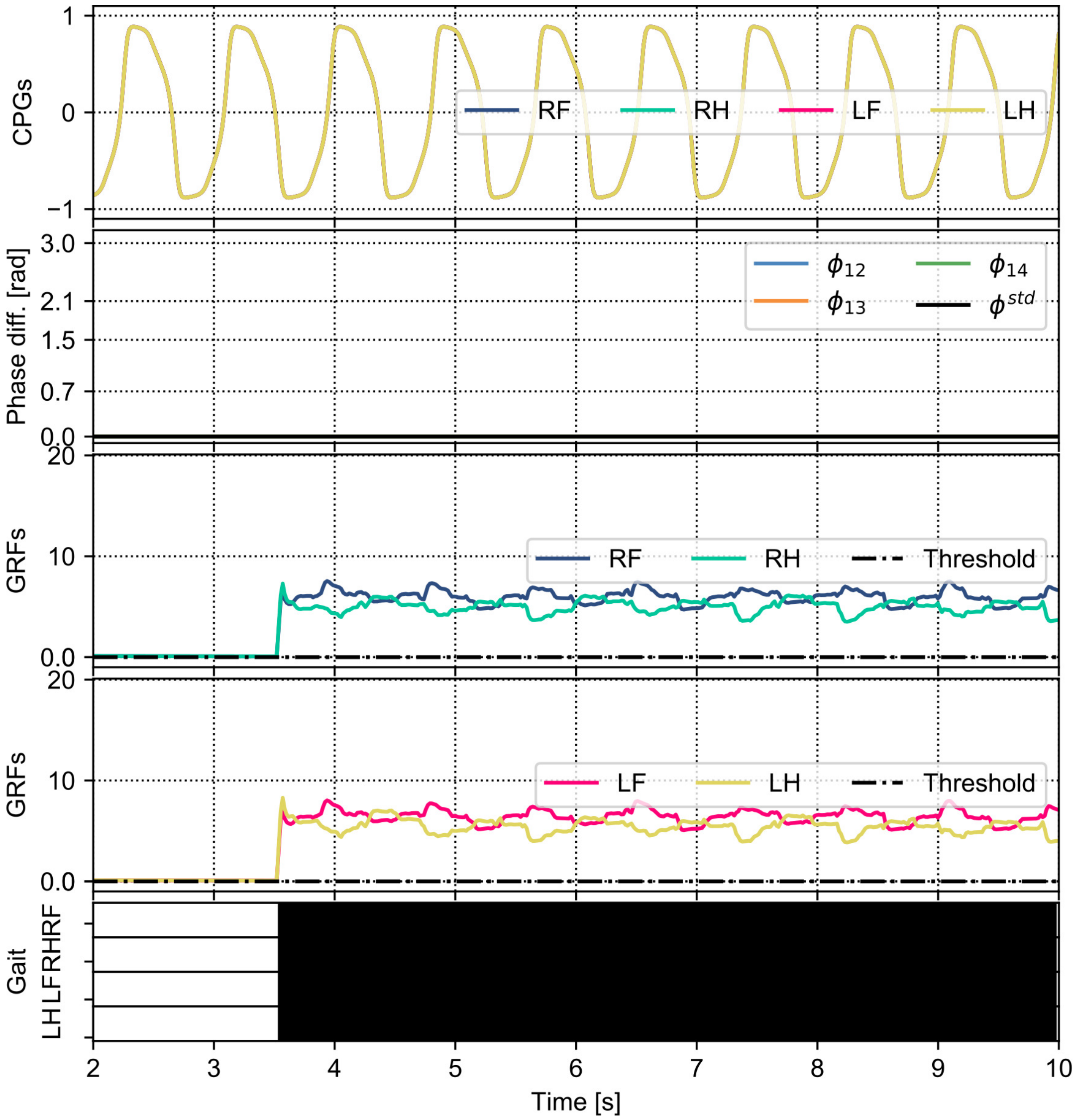

Figure S6. Experimental data of a trial under the PR with $F_t = 0.0$. The sensory feedback was not activated due to the force threshold is zero, thus no phase shift occurred among the CPGs. The phase differences were zeros, the robot four legs always stayed at stance phase without stepping forward.

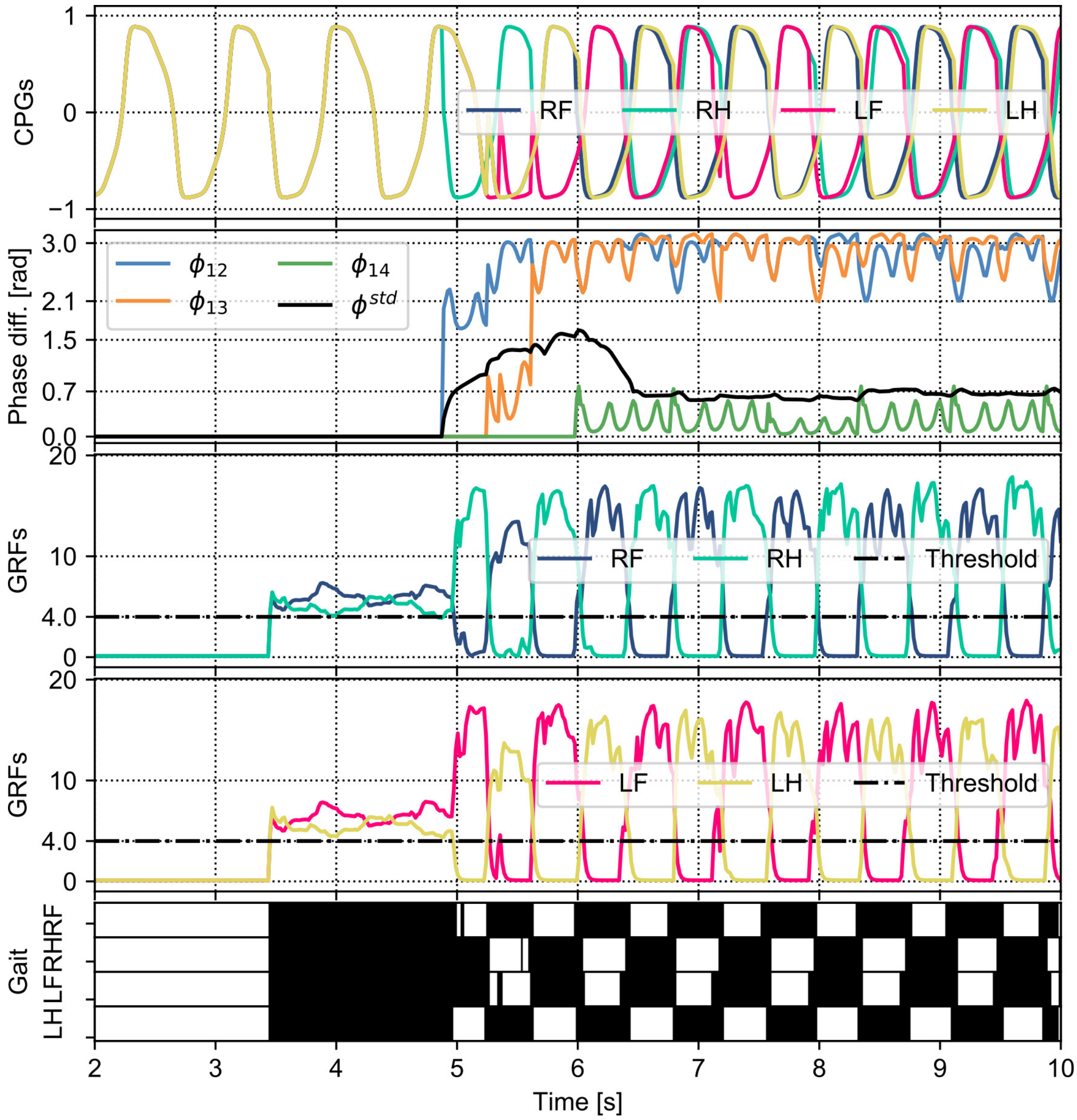

Figure S7. Experimental data of a trial under the PR with $F_t = 0.64$. The sensory feedback with proper force threshold reset CPG phase so that the phase differences converged around (π, π, 0), ϕ^{std} reduced to less than 0.75 after the self-organized robot locomotion emerged.

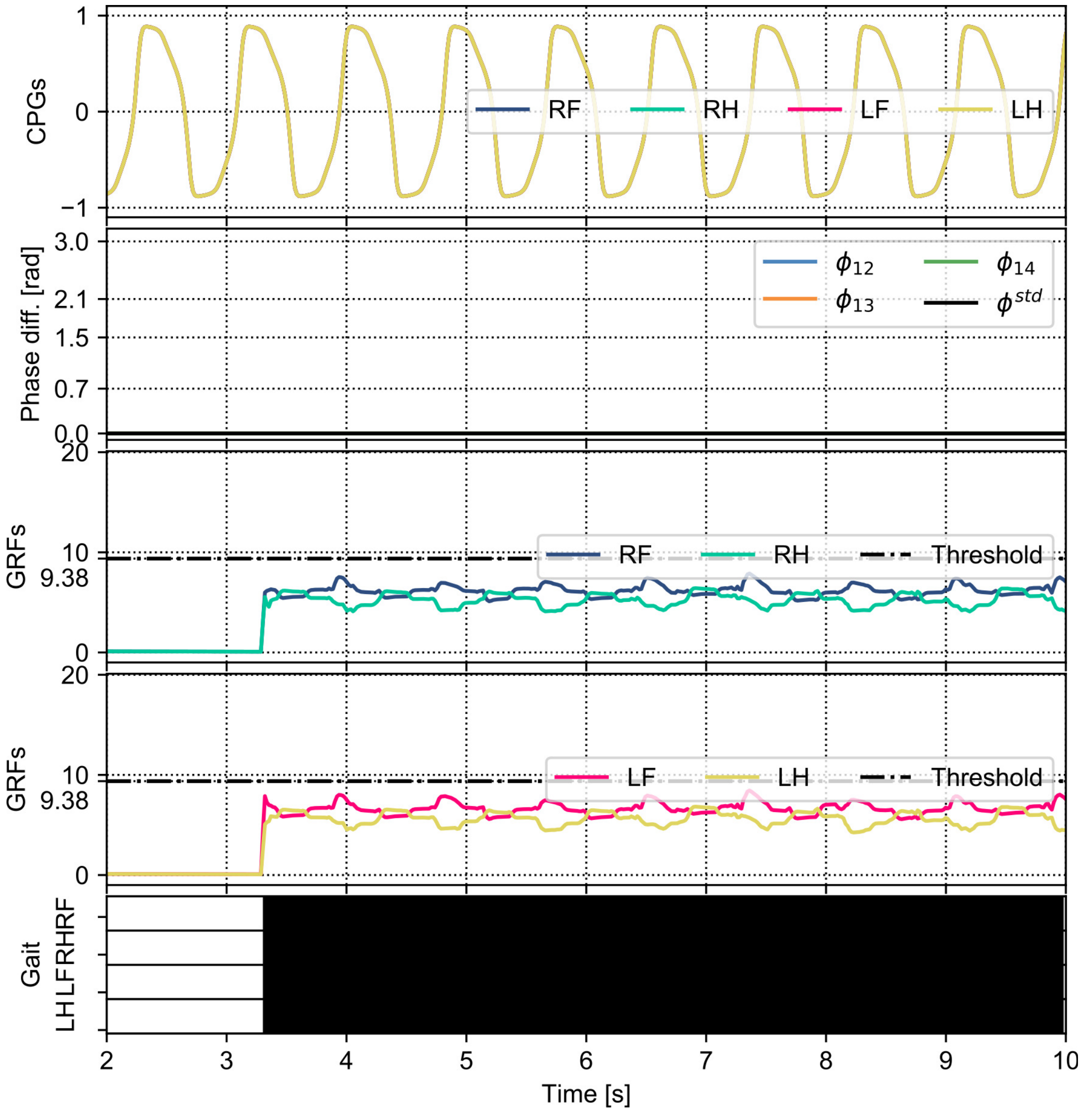

Figure S8. Experimental data of a trial under the PR with $F_t = 1.5$.The sensory feedback has excessive threshold so that the GRFs could not reach the phase resetting condition, thereby no phase shift among the four CPGs.

1.2 Distinctive experimental results of the PR with the same setting and initial conditions

The convergence of the decoupled CPGs with the PR is sensitive to its initial conditions ($o_{ik}(n_0)$, i=1, 2; k=1, 2, 3, 4.) that are CPG outputs at the touch moment. n_0 indicates the moment when the robot was dropped on the ground. Slight differences between the $o_{ik}(n_0)$ may cause the decoupled CPGs different convergent results. Two trials under the PR with same control parameter values ($F_t = 0.45$) and same initialization procedures are demonstrated here, as shown in Figures S9 and S10. The decoupled CPGs with the PR show different results.

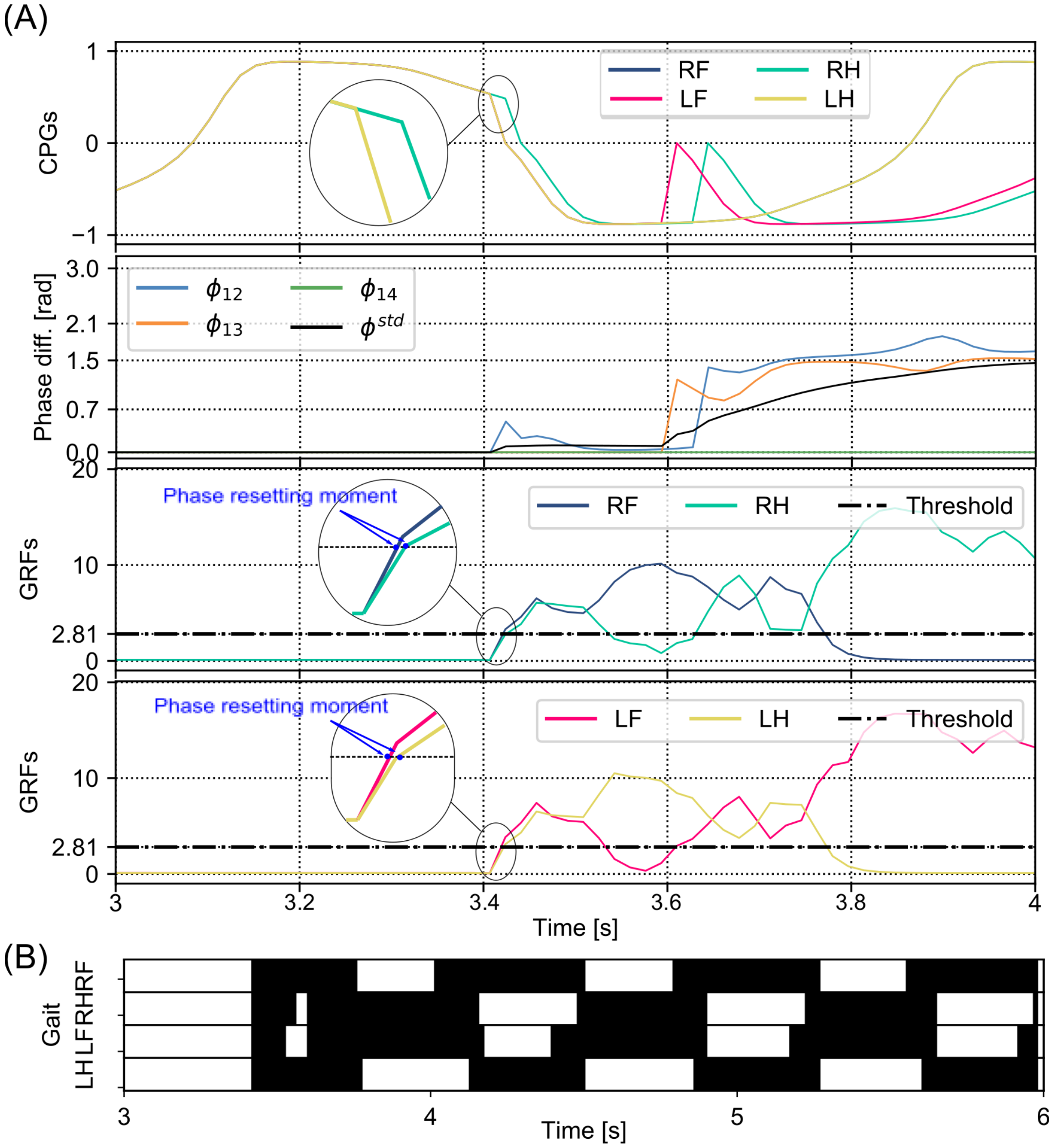

Figure S9. Experimental data of a trial under the PR with $F_t = 0.45$. (A) The four legs' GRFs crossed over the GRF threshold at different moment even the four legs touched the ground at the same moment. As a result, the phases of the four decoupled CPGs were reset at different moment, which leads to the phase differences among them to coordinate leg movements. (B) Consequently, the robot formed a trot gait.

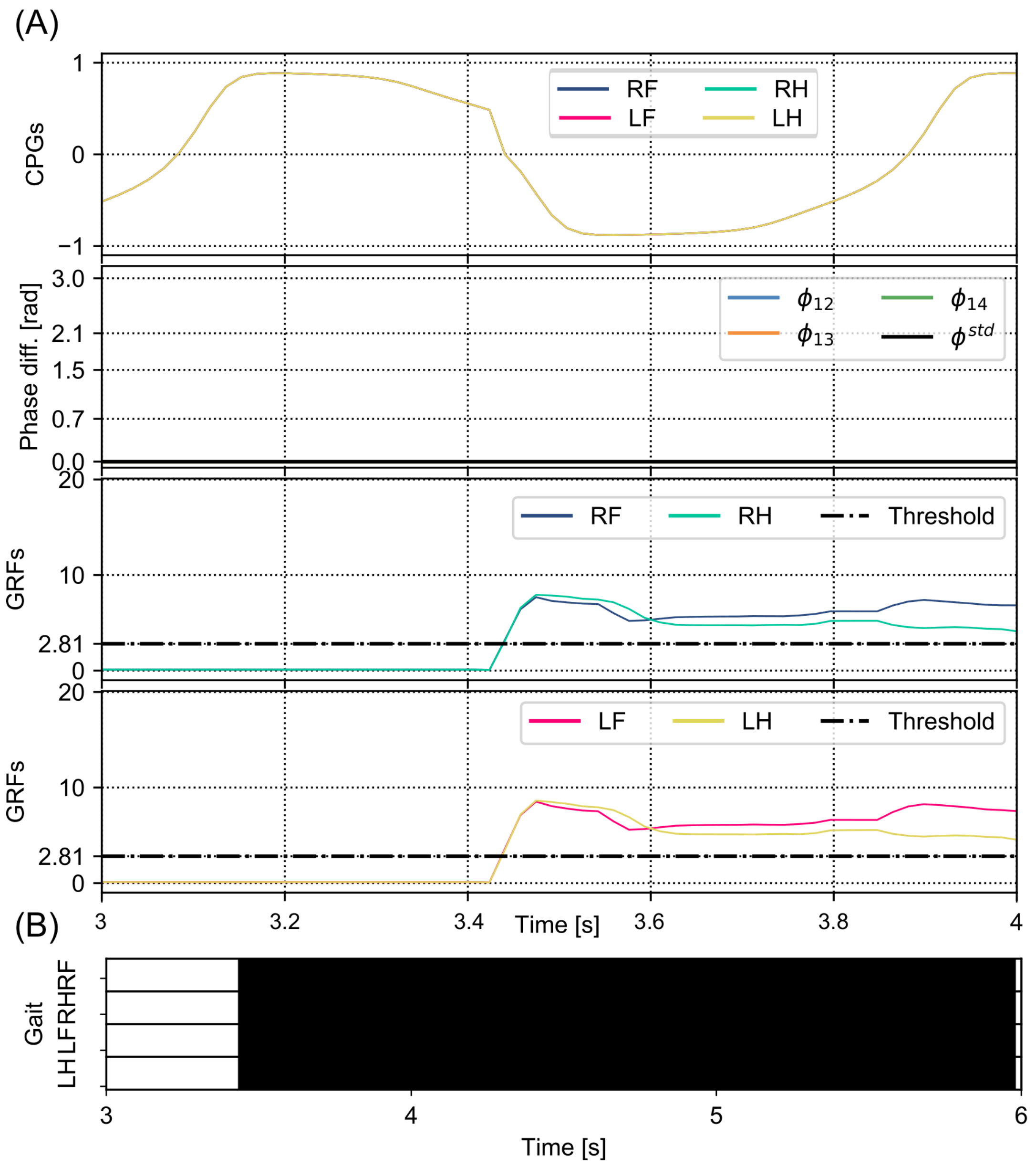

Figure S10. Experimental data of a trial under the PR with $F_t = 0.45$. (A) The four legs' GRFs crossed over the GRF threshold at the same moment, thus their CPG phase were reset at the same moement, thereby no phase differences among them to corrdinate the leg movements. (B) Consequently, the robot did form a gait.

1.3 Experimental data under different situations

This subsection contains eight figures (Figures S11, S12, S13, S14, S15, S16, S17, S18) which demonstrate the experimental data under the PM and PR when the robot encounters the four robot situations. Each figure has five subplots, including 1) the first neuron output ($o_{1k}, k = 1, 2, 3, 4$) of the four neural SO(2)-based CPGs, 2) CPG phase differences (ϕ_{12}, ϕ_{13}, and ϕ_{14}) and the sum standard derivation of the phase differences (ϕ^{std}), 3) the right-side legs' GRF (right front (RF) and right hind (RH) legs), 4) the left-side legs' GRF (left front (LF) and left hind (LH) legs), and 5) gait diagram. In gait diagram, white and black areas represent stance and swing phase, respectively. Note that, the RF, RH, LF, and LH indicates the right front, right hind, left front, and left hind, respectively.

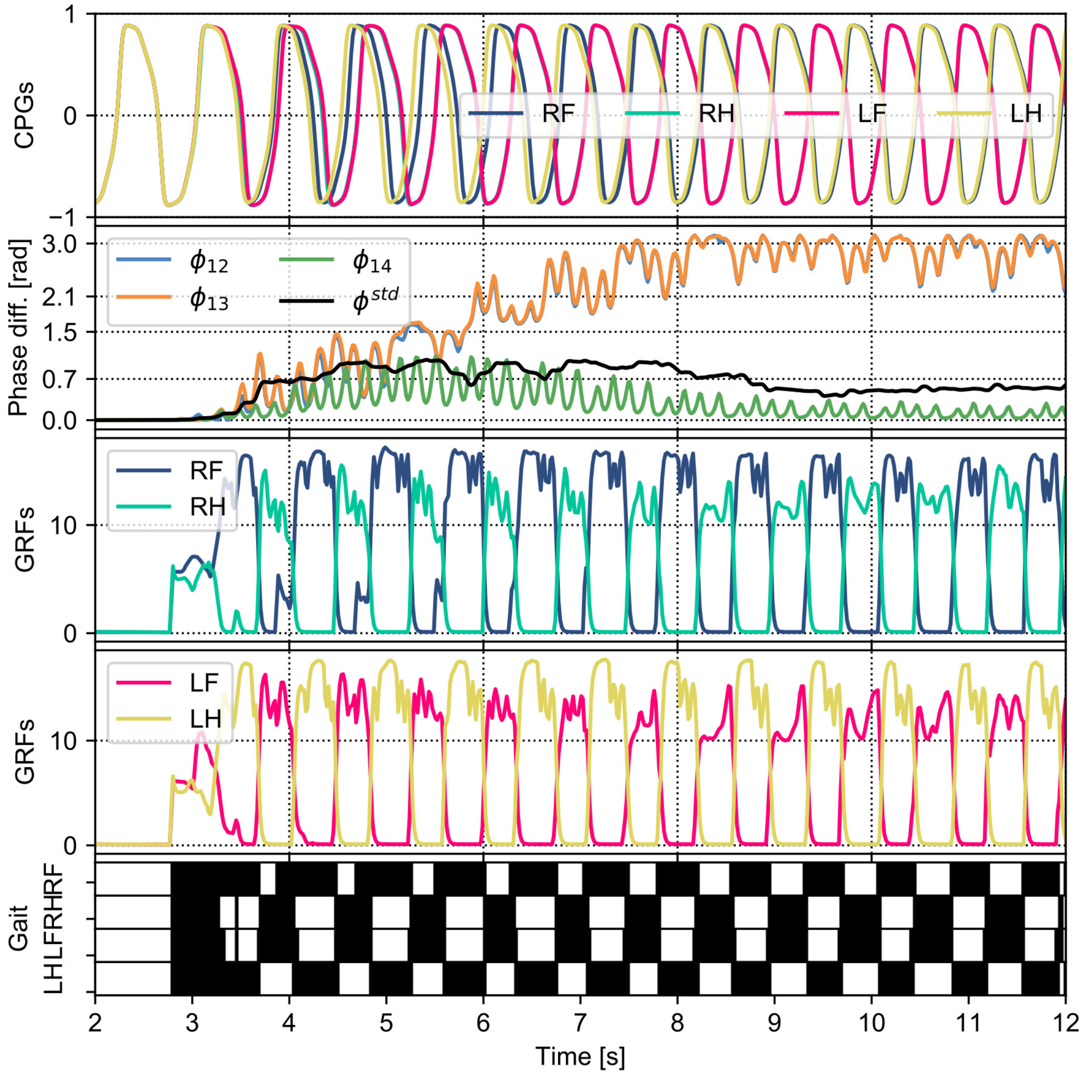

Figure S11. Experimental data of a trial under the PM when the robot was in S1 situation. S1 is a normal situation, as a baseline for comparison with other abnormal situations.

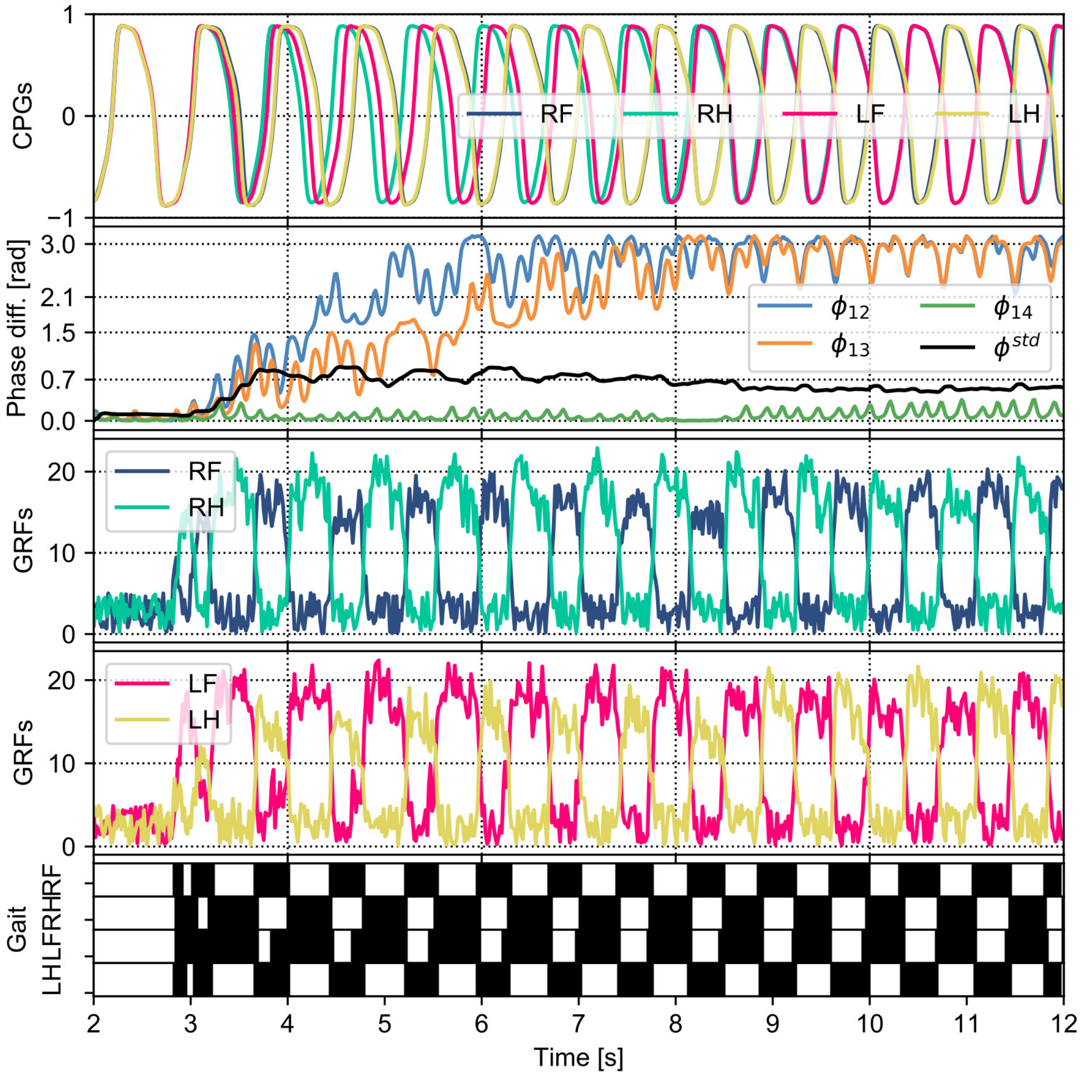

Figure S12. Experimental data of a trial under the PM when the robot was in S2 situation. The GRFs were added Gaussian-distributed noises with a standard deviation of 20% to the maximum GRF amplitude (≈ 5).

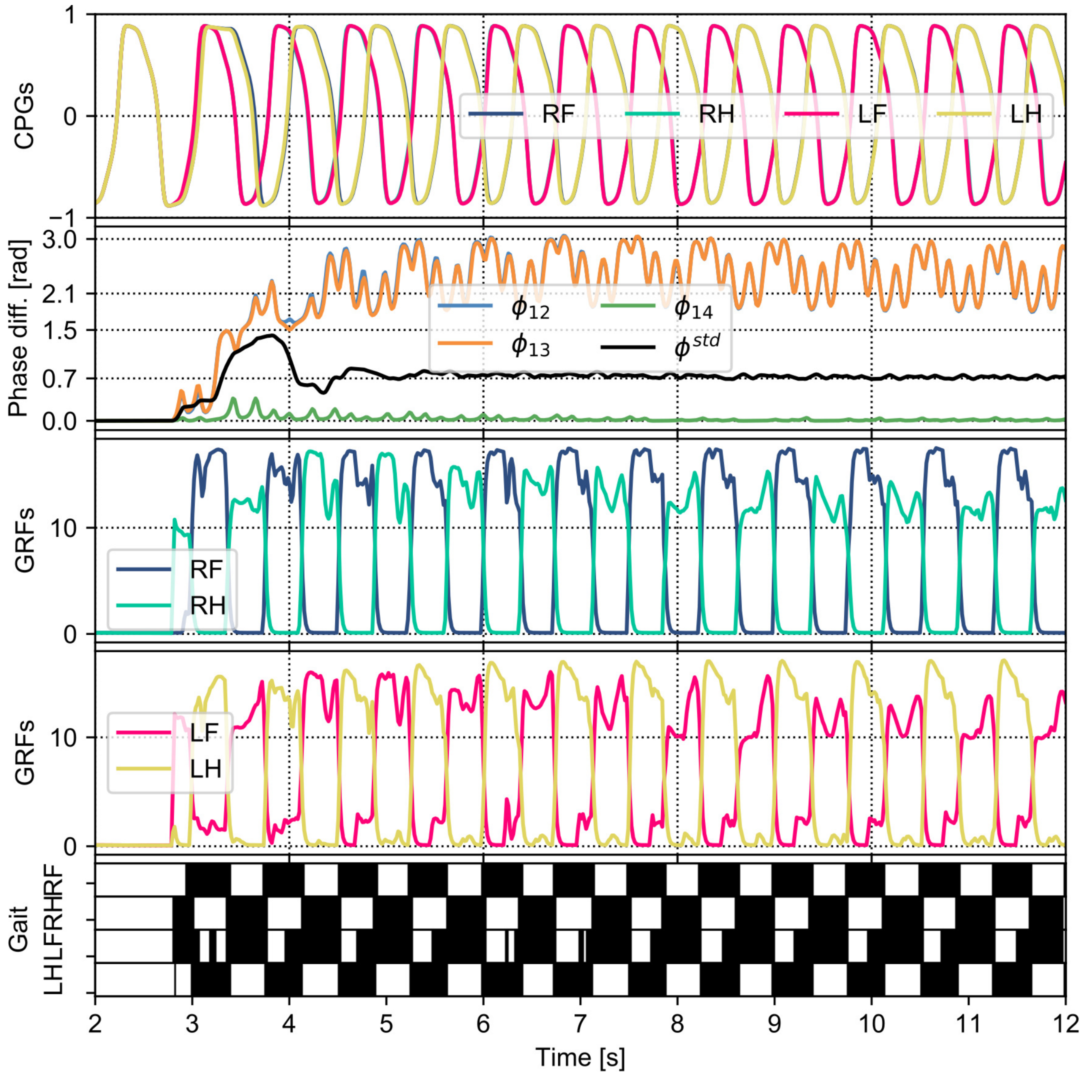

Figure S13. Experimental data of a trial under the PM when the robot was in S3 situation. The right front leg joints were fixed without moving.

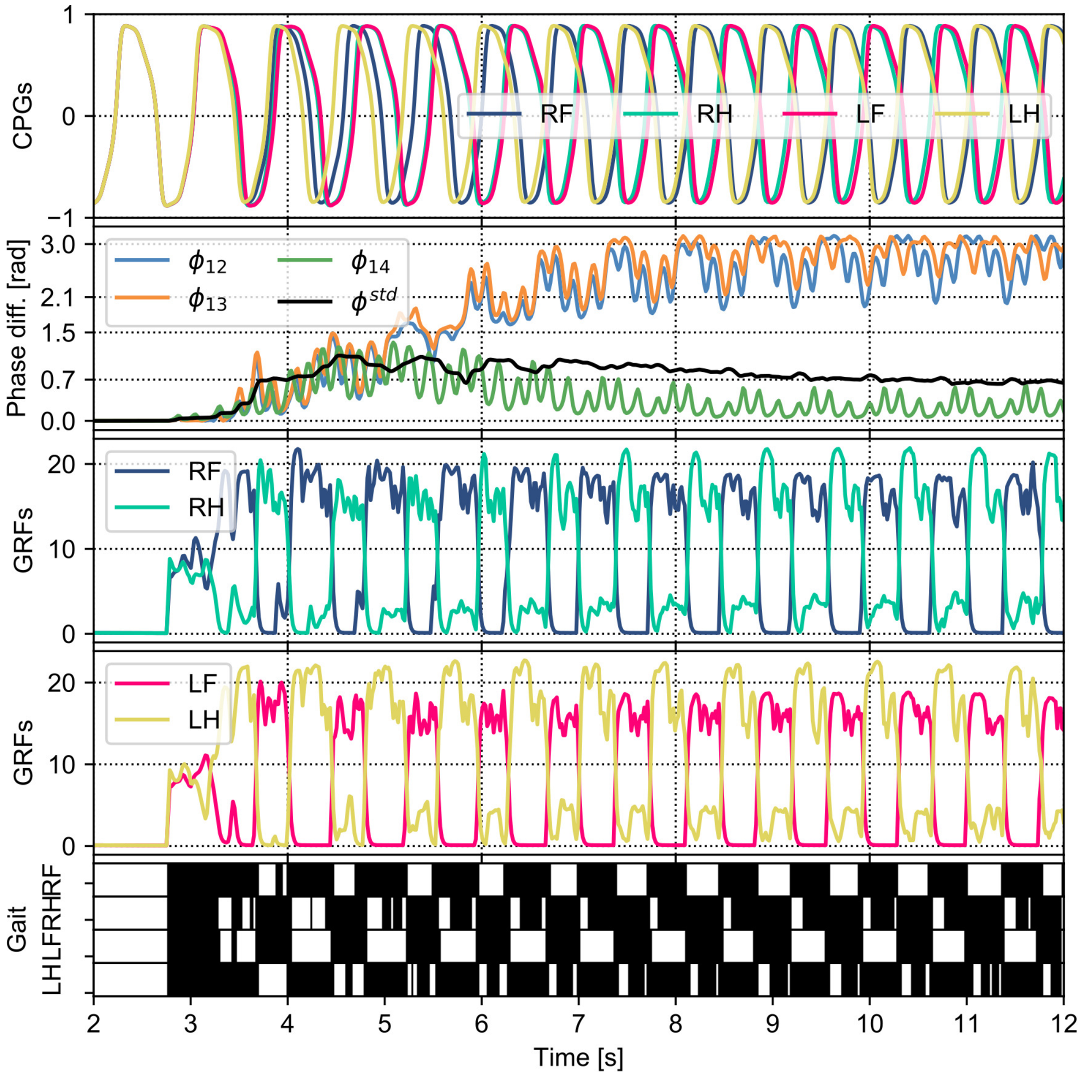

Figure S14. Experimental data of a trial under the PM when the robot was in S4 situation. The rear back was added a payload with 0.6 kg.

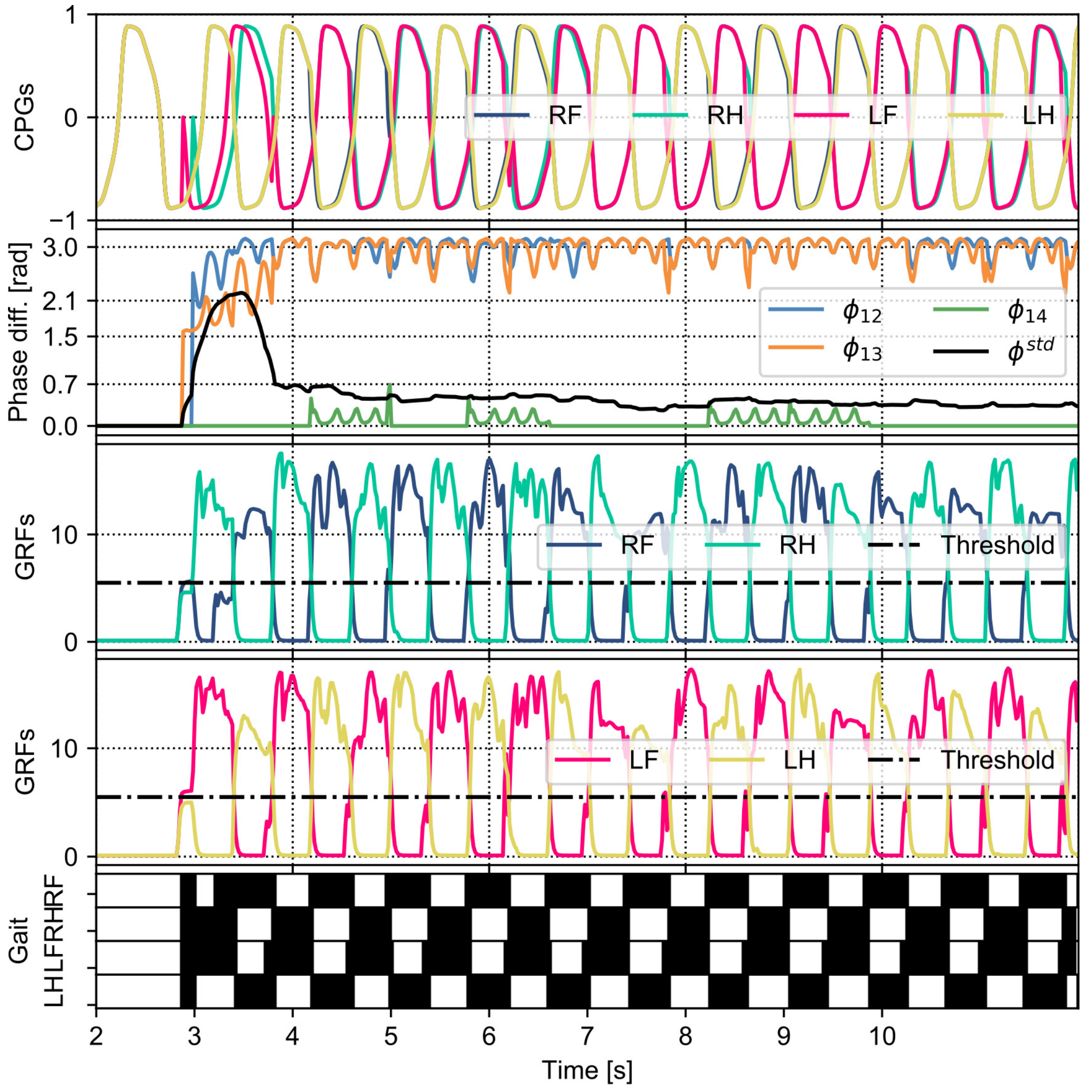

Figure S15. Experimental data of a trial under the PR when the robot was in S1 situation. S1 is a normal situation, as a baseline for comparison with other abnormal situations.

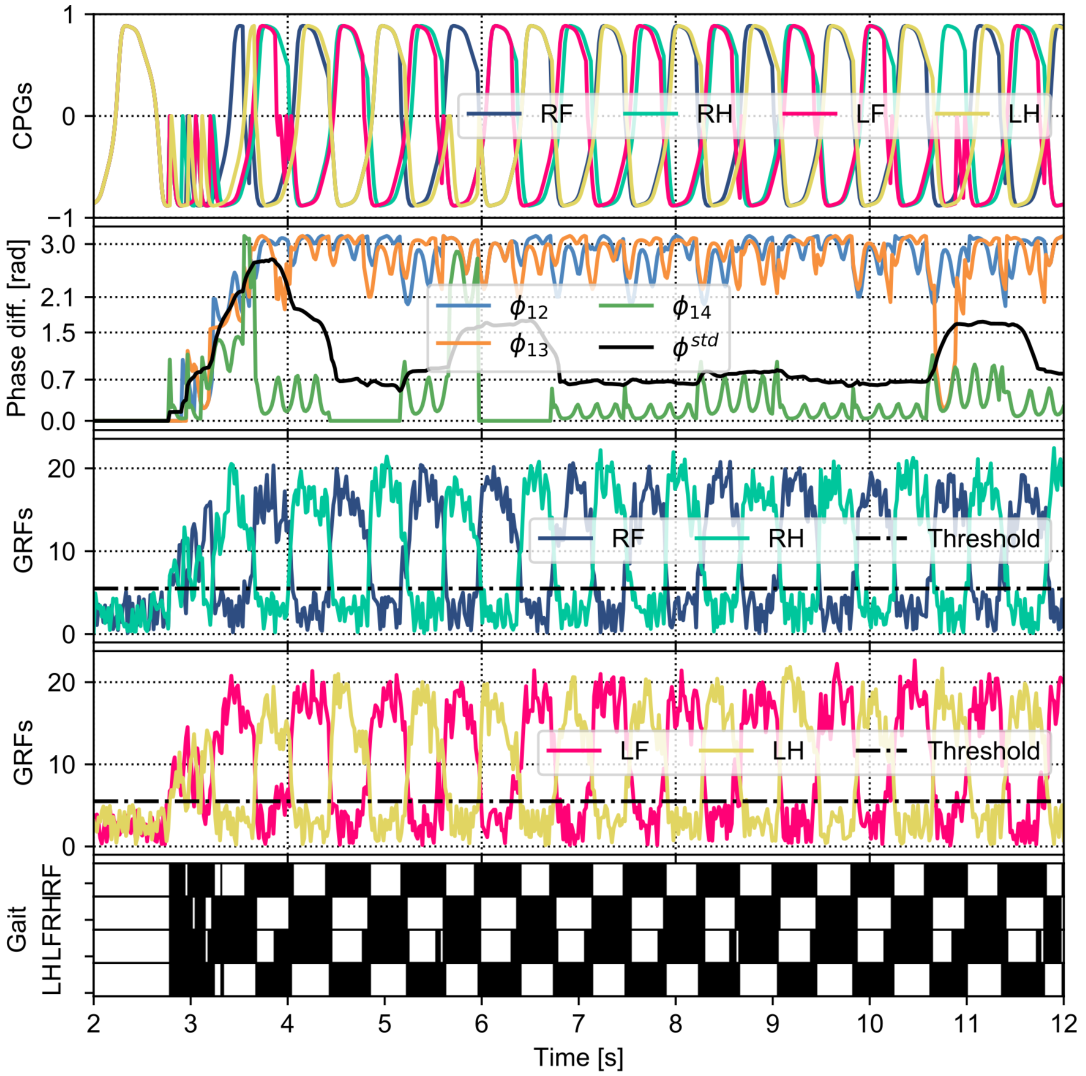

Figure S16. Experimental data of a trial under the PR when the robot was in S2 situation. The GRFs were added Gaussian-distributed noises with a standard deviation of 20% to the maximum GRF amplitude (≈ 5).

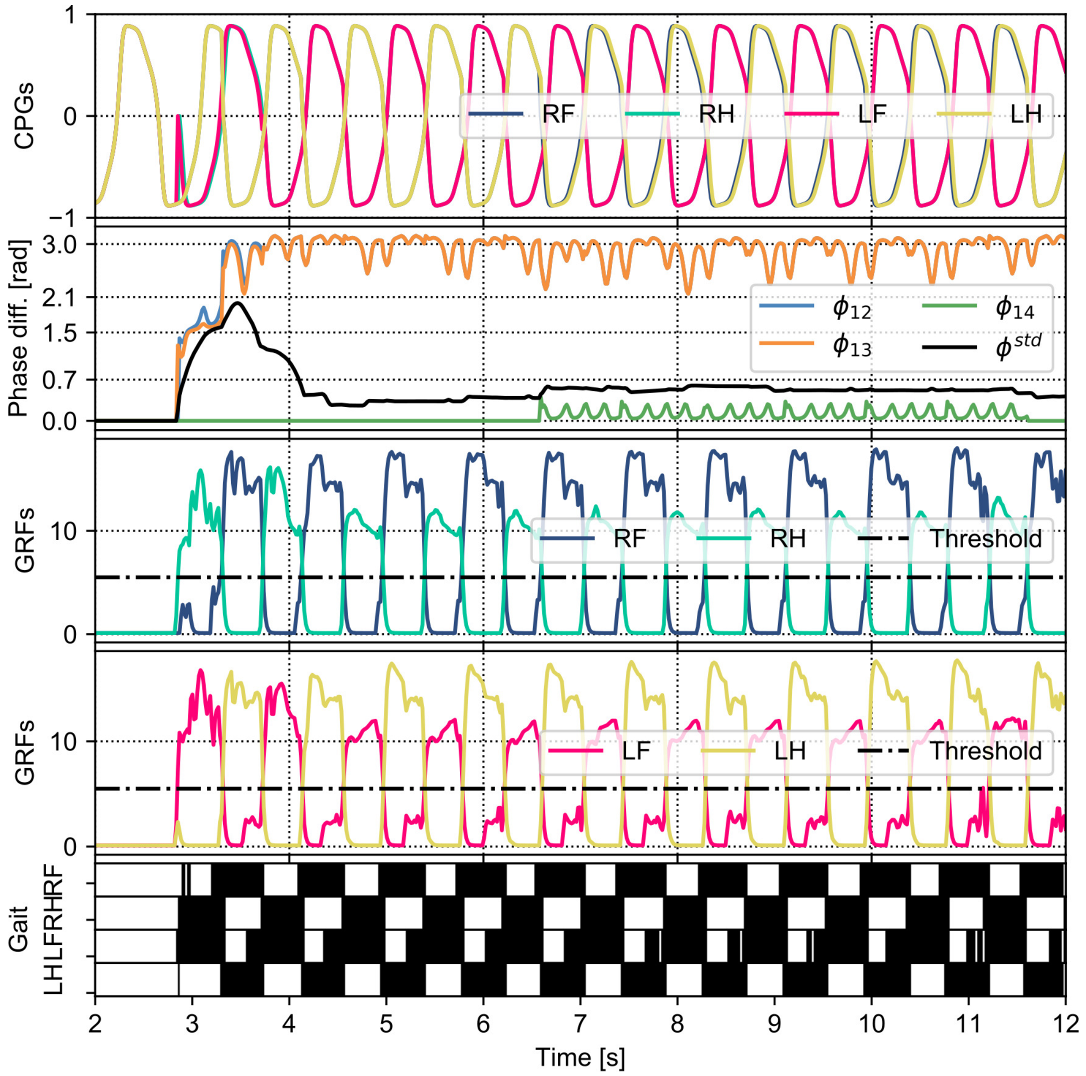

Figure S17. Experimental data of a trial under the PR when the robot was in S3 situation. The right front leg joints were fixed without moving.

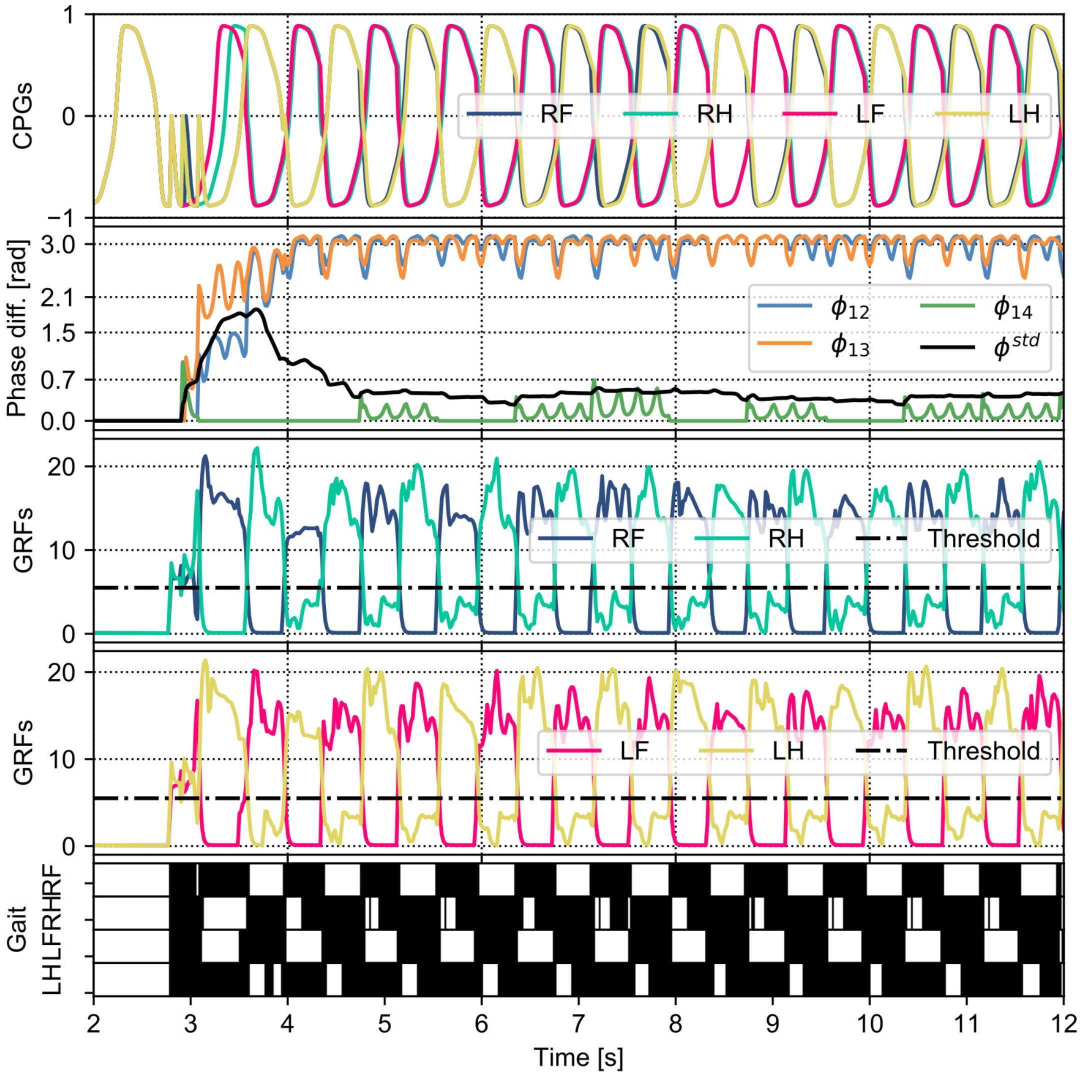

Figure S18. Experimental data of a trial under the PR when the robot was in S4 situation. The rear back was added a payload with 0.6 kg.

2 ANALYSIS OF THE FAILED SELF-ORGANIZED LOCOMOTION

The cases with 0% success rate in Figures 4 and 5 of the main manuscript result from the inappropriate "physical coupling strength" among the CPGs. In this work, the adaptive synchronizations/coordination among the decoupled CPGs are realized via sensory feedback in the form of the PM or PR which provides a physical communication/coupling effects on the CPGs. The PM and PR parameter values (γ of the PM and F_t of the PR) determine the "physical coupling strength". When the parameter values are too small or large, the "physical coupling strength" will be so small or large such that the proper synchronization cannot be achieved. As a result, the CPG phase relationships (ϕ_{12}, ϕ_{13}, and ϕ_{14}) of the decoupled CPGs will not be proper, thus cannot enable the robot to perform a stable gait.

To verify this statement, we have illustrated the phase differences of the decoupled CPGs based on the PM and PR with their improper parameter values, for example, $\gamma = 0$, $\gamma = 1$; $F_t = 0$, $F_t = 1.5$ (see Figures S3, S5, S6, and S8 in the previous section), and implemented the formed phase differences in coupled CPGs-based control on quadruped robots through the following coupling term (Aoi et al., 2010, 2013):

$$f_{ik}(n) = \kappa(n)\xi \sum_{k}^{N} \left(\sin\left(o_{il}(n) - o_{ik}(n) - \phi_{lk}(n)\right)\right), \tag{S1}$$

where $o_{il}(n)$ and $o_{ik}(n)$ are the outputs of the ith neurons in CPGs l and k. ϕ_{lk} is the phase differences of CPG k with respect to CPG l. ξ is a communication gain. We empirically set it to 0.01.

From the experimental results, shown in Figures S3, S5, S6, and S8, we summarize that the phase differences of the CPGs under the improper parameter values are zero, thus we can set:

$$\Phi = \begin{pmatrix} 0 & 0 & 0 & 0 \\ 0 & 0 & 0 & 0 \\ 0 & 0 & 0 & 0 \\ 0 & 0 & 0 & 0 \end{pmatrix} \tag{S2}$$

Replacing this phase differences into coupled CPG-based control (Equation (S1)), we employ this control to the quadruped robot, the experimental results shown in Figure S19. From this, one can find that the robot did not generate a gait even based on the coupled CPGs. It is similar with the results based on the decoupled CPGs with improper parameter values. This suggests that the reason of the cases with 0% success rate is that the "physical coupling strength", formed by the PM/PR, is not proper.

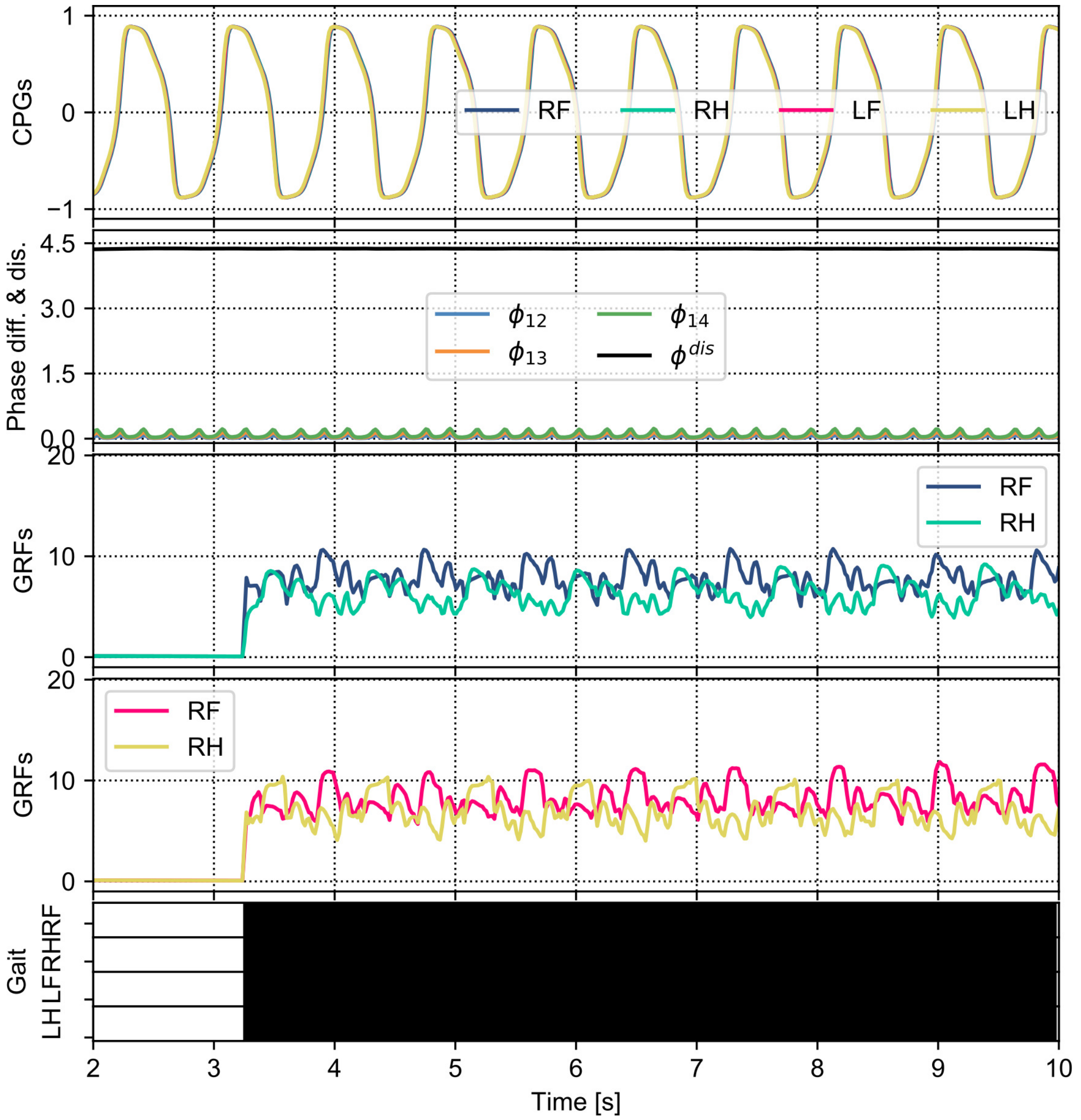

Figure S19. Experimental data of a trial under the coupled CPG-based control. The coupling strength was predefined by the phase differences generated in a trial under decoupled CPG-based control with improper parameter values. The robot could not generate self-organized locomotion, the four legs stays at stance phase. This results are similar with the decoupled CPGs with improper parameter values (Figures S3, S5, S6, and S8).

REFERENCES

Aoi, S., Katayama, D., Fujiki, S., Tomita, N., Funato, T., Yamashita, T., et al. (2013). A stability-based mechanism for hysteresis in the walk–trot transition in quadruped locomotion. *Journal of The Royal Society Interface* 10, 20120908

Aoi, S., Yamashita, T., Ichikawa, A., and Tsuchiya, K. (2010). Hysteresis in gait transition induced by changing waist joint stiffness of a quadruped robot driven by nonlinear oscillators with phase resetting. In *2010 IEEE/RSJ International Conference on Intelligent Robots and Systems* (IEEE), 1915–1920
